# Galectin-3 shapes toxic alpha-synuclein strains in Parkinson’s disease

**DOI:** 10.1007/s00401-023-02585-x

**Published:** 2023-05-18

**Authors:** Juan García-Revilla, Antonio Boza-Serrano, Yiyun Jin, Devkee M. Vadukul, Jesús Soldán-Hidalgo, Lluís Camprubí-Ferrer, Marta García-Cruzado, Isak Martinsson, Oxana Klementieva, Rocío Ruiz, Francesco A. Aprile, Tomas Deierborg, José Luis Venero

**Affiliations:** 1 Instituto de Biomedicina de Sevilla (IBiS), Hospital Universitario Virgen del Rocío/CSIC, Universidad de Sevilla, Seville, Spain; 2 Departamento de Bioquímica y Biología Molecular, Facultad de Farmacia, Universidad de Sevilla, Seville, Spain; 3 Experimental Neuroinflammation Laboratory, Department of Experimental Medical Science, Lund University, BMC B11, 221 84 Lund, Sweden; 4 Department of Chemistry, Molecular Sciences Research Hub, Imperial College London, London, W12 0BZ UK; 5 Institute of Chemical Biology, Molecular Sciences Research Hub, Imperial College London, London, W12 0BZ UK; 6 Medical Microspecroscopy Lab, Department of Experimental Medical Science, SRA: NanoLund, Multipark, Lund University, BMC B10, 221 84 Lund, Sweden

**Keywords:** Parkinson’s disease (PD), Galectin-3 (GAL3), α-synuclein (αSYN), Lewy body (LB)

## Abstract

Parkinson’s Disease (PD) is a neurodegenerative and progressive disorder characterised by intracytoplasmic inclusions called Lewy bodies (LB) and degeneration of dopaminergic neurons in the substantia nigra (SN). Aggregated α-synuclein (αSYN) is known to be the main component of the LB. It has also been reported to interact with several proteins and organelles. Galectin-3 (GAL3) is known to have a detrimental function in neurodegenerative diseases. It is a galactose-binding protein without known catalytic activity and is expressed mainly by activated microglial cells in the central nervous system (CNS). GAL3 has been previously found in the outer layer of the LB in post-mortem brains. However, the role of GAL3 in PD is yet to be elucidated. In post-mortem samples, we identified an association between GAL3 and LB in all the PD subjects studied. GAL3 was linked to less αSYN in the LB outer layer and other αSYN deposits, including pale bodies. GAL3 was also associated with disrupted lysosomes. In vitro studies demonstrate that exogenous recombinant Gal3 is internalised by neuronal cell lines and primary neurons where it interacts with endogenous αSyn fibrils. In addition, aggregation experiments show that Gal3 affects spatial propagation and the stability of pre-formed αSyn fibrils resulting in short, amorphous toxic strains. To further investigate these observations in vivo, we take advantage of WT and Gal3KO mice subjected to intranigral injection of adenovirus overexpressing human αSyn as a PD model. In line with our in vitro studies*,* under these conditions, genetic deletion of GAL3 leads to increased intracellular αSyn accumulation within dopaminergic neurons and remarkably preserved dopaminergic integrity and motor function. Overall, our data suggest a prominent role for GAL3 in the aggregation process of αSYN and LB formation, leading to the production of short species to the detriment of larger strains which triggers neuronal degeneration in a mouse model of PD.

**Supplementary Information:**

The online version contains supplementary material available at 10.1007/s00401-023-02585-x.

## Introduction

Parkinson’s disease (PD) is the second most prevalent neurodegenerative disease in the world [26]. It is characterised by intense neurodegeneration in the basal ganglia area leading to severe and progressive motor impairment. The presence of Lewy bodies (LBs), described as neuronal intracytoplasmic deposits of α-synuclein (αSYN), is the second hallmark of the disease [25]. While dopaminergic neurons from the substantia nigra (SN) are frequently the most affected cells, neurodegeneration and LB formation commonly appear in other central and enteric nervous system locations even years before than in the SN [26].

The mechanisms connecting αSyn self-assembly and neurodegeneration are currently under investigation. However, several hypotheses have been proposed, including genetic factors, prion-like spreading of αSyn, mitochondrial damage or environmental factors [31]. A prominent role for neuroinflammation has also been suggested, as post-mortem analyses often show clear signs of microglia activation [41]. While excessive and sustained production of cytokines is known to trigger neurodegeneration, its link with αSyn aggregation is still largely uncharacterised. Microglial cells are considered the driving factor in neuroinflammatory conditions and the primary source of pro-inflammatory molecules in the central nervous system (CNS). Over the past years, multiple microglia phenotypes have been described in distinct neurodegenerative diseases and conditions [55]. However, a specific PD microglial phenotype has not been elucidated yet. One of the most upregulated markers in neurodegenerative diseases is galectin-3 (GAL3) [37]. GAL3 is a galactose-binding protein without known catalytic activity and is expressed mainly by activated microglial cells in the central nervous system (CNS). We have previously described how GAL3 can be released by microglial cells in neuroinflammatory conditions and interact with different microglial receptors like Tlr4 [10] and Trem2 [7]. Moreover, microglial GAL3 can be upregulated in the SN in an MPTP model of PD [21], supporting the idea of a potential PD-specific phenotype.

In our previous study, genetic deletion of GAL3 not only decreased microglia reactivity but also improved cognitive and behavioural status in a mice model of AD [7]. However, several studies have revealed non-inflammatory roles for GAL3 that could place this molecule as a node between inflammation and αSyn deposition. For instance, Flavin and colleagues [20] described GAL3 in the outer layer of LB in PD patients as potentially associated with vesicle rupture. However, it is unknown if the presence of GAL3 in the LB outer range is causally related to PD or if it is a secondary event related to the heterogeneous nature of LB, such as the recruitment of organelles and organelles damage. While GAL3 is known to efficiently bind the highly glycosylated inner membrane of lysosomes, it has been proposed that GAL3 could be part of a molecular platform implicated in repairing broken lysosomes [30]. Nevertheless, the interaction of GAL3 and αSyn and the effect on αSyn aggregation have not been described, and hence, a role in pathological αSyn aggregation should not be discarded. Indeed, the identification of endogenous molecules playing significant roles in αSyn aggregation associated with LB formation is of paramount importance. Supporting a pathological role of GAL3 in PD pathogenesis, recent GWAS studies have identified *LGALS3* as a genetic risk for PD [4]. In addition, serum levels of GAL3 levels have been found elevated in PD patients [13, 64], and interestingly, a precise correlation between GAL3 serum levels and disease progression (Hoehn and Yahr scores) was recently demonstrated [62].

Here we investigated whether GAL3 is associated with pathological αSyn strains and whether this association may drive different stages of LB pathology. In our work, we demonstrate that: (i) GAL3 is present in different αSYN deposits from pale bodies to LB in brains from deceased PD patients, (ii) GAL3 is inherently associated with both αSYN strains and disrupted lysosomes, (iii) GAL3 is present in low levels in mice and human brain in sufficient amount for neuronal internalisation, (iv) Gal3 prevents αSyn aggregation in vitro and disrupts pre-formed fibrils resulting in short, amorphous toxic strains, and (v) in vivo experiments with long-term overexpression of human αSyn in the ventral mesencephalon, Gal3KO mice presented major rounded intracytoplasmic inclusions of αSyn in the ventral mesencephalon resembling human LB, better motor performance and complete preservation of nigral dopaminergic neurons. Overall, our data suggests a prominent endogenous role for GAL3 in pathological αSYN strains associated with LB formation and toxicity, thus pointing to GAL3 inhibition as a potential future therapeutical strategy to prevent or slow down PD progression.

## Methods

### Animals

All procedures were performed according to the Spanish (RD 53/2013) and European (86/609/EU) regulations and their use was approved by the ethical committee from the University of Seville.

Galectin-3 knockout (−/−) transgenic mice have a C57BL/6 background and were originally obtained from Dr K. Sävman at Gothenburg University (Sweden). C57BL/6 wild-type mice were obtained from the Center of Production and Animal Experimentation at the University of Seville, where all animals were maintained in.

Animals were kept at constant temperature (22 ℃) and humidity (60%) with free access to food and water and a 12 h dark/12 h light cycle.

### Genotyping

The genotype of mice was determined in a two-step protocol. First, DNA was extracted using QuickExtract™ DNA Extraction Solution 1.0 (Epicentre, Madison, WI, USA). According to the manufacturer’s protocol, samples were immersed in the solution and vigorously shaken for 15 s. After agitation, samples were incubated for 15 min at 65 ℃, followed by another 15 s agitation, and finally, 2 min incubation at 68 ℃. Second, MyTaq™ Red DNA Polymerase (Bioline, London, UK) kit was then employed along with self-designed primers: *Gal3*-common CTACTCCTTGGCCCTCTAGGTC-3′, *Gal3*-WT TGA AAT ACT TAC CGA AAA GCT GTC TGC -3′ (single 490 bp band) and *Gal3*-KO GCTTTTCTGGATTCATCGACTGTGG-3′ (single 316 bp band). Briefly, samples were heated to 94 °C for 1 min before performing 35 cycles of the following three steps: denaturation at 95 °C for 15 s, an annealing stage consisting of 15 s at 58 ℃ and an elongation step at 72 ℃ for 10 s. PCR products were separated by electrophoresis in 1.5% agarose gel and visualized with the help of the DNA intercalating agent RedSafe™ Nucleic Acid Staining Solution (iNtRON Biotechnology, Seongnam, South Korea) under UV light.

### Intracranial injection

12–14 weeks-old mice were anaesthetised with a mixture of ketamine (50 mg/kg, Ketamidor^®^, Richter Pharma, Wels, Austria) and medetomidine (10 mg/kg, Domtor^®^, Ecuphar, Oostkamp, Belgium) intraperitoneally (i.p.) and given pre-emptively analgesia with buprenorphine (0.05 mg/kg, Bupaq^®^, Richter Pharma, Wels, Austria) subcutaneously (s.c.). Mice were placed into a stereotaxic device (Harvard Apparatus, Holliston, MA, USA) and secured with mice-adapted teeth and ear bars. Ophthalmic gel drops were applied to prevent eyes from drying, and anaesthesia-associated hypothermia was prevented with a heat source.

First, the head was aseptically cleaned with 70% ethanol. A midline incision was made to expose the skull. Injection site coordinates (+ 1.2 mm anterior, − 1.3 mm lateral from lambda) were previously determined by experimental procedures. An approximately 1 mm diameter craniotomy was made over the brain’s left hemisphere using a 0.8 mm diameter drill bit connected to a hand-held dental drill. Skull remains were carefully removed to avoid duramater puncture.

The injection was carried out using a 10-µl Hamilton^®^ 701N syringe with a borosilicate capillary glass with an approximate outer diameter of 70 µm (Harvard Apparatus, Holliston, MA, USA) attached to the tip to minimize inflammatory response derived from injection trauma. The tip was then moved to the ventral coordinate (− 4.2 mm from duramater), and 1.5 µl of the viral vector (1.0 × 10^13^ vg/ml) was slowly injected at 0.25 µl/min. AAV5-CBA-GFP, overexpressing the green fluorescent protein (further referred as AAV5-GFP), and AAV5-CBA-αSYN, overexpressing the human form of α-synuclein (further referred as AAV5-hSYN), were obtained from *Michael J. Fox Foundation for Parkinson’s Research* in collaboration with UNC Vector Core (Chapel Hill, NC, USA). Once the surgery was finalized and the skin sutured, mice received a 50-µl subcutaneous injection of 0.5 mg/ml Atipamezol (Nosedorm^®^, Karizoo, Barcelona, Spain) to antagonise the anaesthetic effect.

### Behavioural tests

Behavioural tests were all performed in the Faculty of Pharmacy at the University of Seville. Animals were placed in the behavioural room for at least 1 h prior experiment to habituate them to the new environment. Mice were housed individually the week before to start the tests. Mice were distributed into four groups according to injections. WT injected with AAV5-GFP (further referred as WT GFP, *n* = 14), WT injected with AAV5-hSYN (further referred as WT hSYN, *n* = 14), Gal3KO injected with AAV5-GFP (further referred as Gal3KO GFP, *n* = 13) and Gal3KO injected with AAV5-hSYN (further referred as Gal3KO hSYN, *n* = 14) were tested 8 weeks and 24 weeks after injection.

#### Rotarod

Mice were first habituated to the rotarod apparatus (Ugo Basile, Gemonio, Italy) with a 5-min session at a constant speed of 5 rpm. To test motor coordination, animals faced four sessions at constant acceleration from 5 to 40 rpm for 5 min. Mice took a 15-min break between each session. The day after, animals faced another 5-min session at constant acceleration to evaluate coordination memory and learning ability. When an animal fell off the rotarod, it was re-located in the roller by the experimenter. The number of total falls in each session was measured. Sessions were recorded for later analysis.

#### Cylinder test

Lateralisation of motor capacity was measured contra and ipsilaterally by the cylinder test. For that, mice were placed in a 15-cm diameter, 30 cm-high glass cylinder for 5 min without previous habituation. Because of natural explorative behaviour, mice inspect the cylinder by touching the glass walls. The number of times the mice touched the glass with each front paw was counted and a ratio between the right and left paw was measured. Sessions were recorded for later analysis.

### Protein purification

Recombinant α-synuclein (αSyn) in the expression vector pT7-7 (Addgene, Watertown, NY, United States), gifted from Hilal Lashuel [45], was transformed into BL21-Gold (DE3) *E. coli*, expressed and purified as previously reported [3]. To assess the purity of αSyn and to ensure the protein was free from any post-translational modifications, 50 µM αSyn in HPLC grade water was mixed with acetonitrile and analyzed by ES-LC MS. This was performed using the Mass Spectrometry facilities available at the Department of Chemistry, Molecular Science Research Hub, Imperial college London.

Recombinant galectin-3 (Gal3) was produced by the Lund-Protein Production Platform (Lund University, Sweden). Briefly, Gal3 production was performed in strain *E. coli* TUNER(DE3)/pET3c-hum-Gal3 grown in LB medium, 18 ℃, 250 rpm with 1 mM IPTG overnight. After cell lysis and ultracentrifugation, Gal3 was purified on a 20-ml lactocyl-sepharose column. Peak fractions containing Gal3 were pooled and dialyzed against phosphate buffer saline (PBS, Nzytech, Lisbon, Portugal), pH 7.4.

### Aggregation assays

αSyn seeded aggregation was monitored as previously reported [2]. αSyn pre-formed fibrils (PFFs) were prepared as follows: First-generation fibrils were generated by incubating 300 µL of 70 µM monomeric αSyn in PBS (with 0.01% NaN_3_ to prevent bacterial growth) at 37 ℃ for 4 days, shaking at 200 rpm. The resulting fibrils were centrifuged at 16900 g for 30 min; the pellet was washed twice and resuspended in 300 µL of PBS and then sonicated for 1 min at 10% maximum power with 0.3 s on/0.7 s off cycles using a probe sonicator (Bandelin, Sonoplus HD 2070). Second-generation fibrils, i.e., PFFs, were formed by incubating 100 µM monomeric αSyn with 10 µM first-generation fibrils in 500 µL of PBS and 0.01% NaN_3_ (Sigma Aldrich, St Louis, MO, USA) for 13–14 h in quiescence. Then, they were sonicated at 10% power, 0.3 s on/0.7 s off cycles, for 20 s. The concentration of first and second generation fibrils was determined by incubating an alquot of fibrils in 4 M guanidine hydrochloride for 1 h at room temperature (RT) and measuring the absorbance of the solution at 275 nm using a Cary 60 UV–vis spectrophotometer (Agilent Technologies, Santa Clara, CA, USA), ε275 nm = 5600 M^–1^ cm^–1^. 4 µM PFFs were incubated in the presence of 165 µM recombinant Gal3 variants in PBS for 2 h at RT. This solution was then supplemented with monomeric αSyn and thioflavin-T (ThT), resulting in final concentrations of 1 µM αSyn PFFs, 40 µM Gal3 variants, 20 µM monomeric αSyn, and 20 µM ThT. Aggregation assays were performed in quiescence at 37 ℃. Control experiments were performed by preparing the same samples where PBS was used in place of Gal3. For monitoring the remodelling of αSyn fibrils, 10 µM αSyn PFFs were incubated in the presence of the 20 µM Gal3 variants in PBS and either 20 µM ThT or 30 µM 1-aniline-8-naphthalene sulfonate (ANS) under quiescent conditions at 37 ℃.

Plates were sealed to prevent evaporation. ThT emission was monitored at 480 nm upon excitation at 440 nm; ANS emission was monitored at 480 nm upon excitation at 380 nm using a CLARIOstar Plus plate reader (BMG Labtech, Allmendgruen, Germany).

#### Native PAGE

10 µM αSyn PFF was incubated in the absence or presence of 20 µM Gal3 in PBS in quiescence overnight at 37 ℃. After incubation, the insoluble protein fraction was separated by centrifugation at maximum speed (16900 g for 30 min) using a benchtop centrifuge and loaded on Native PAGE (Thermo Fisher Scientific, Walthan, MA, USA) according to the manufacturer's instructions. The samples were diluted at a 1:1 ratio in 2X Native PAGE sample buffer (100 mM Tris–HCl, 100% Glycerol, 0.00025% Bromophenol blue, pH 8.6) and were transferred onto a 0.45-µm nitrocellulose membrane for 7 min at 20 V with the i-Blot 2 (Thermo Fisher Scientific). The membrane was later blocked in PBS-0.1% Tween (PBS-Tw) and 5% non-fat milk overnight at 4 ℃ under constant shaking. The membrane was then incubated overnight in 1:1000 anti-αSyn antibody (Abcam) in PBS-Tw at 4 ℃ under constant shaking. Later, the membrane was washed 3 times in PBS-Tw for 10 min and then incubated in 1:5000 Alexa Fluor 555 goat anti-rabbit IgG (H + L) (Invitrogen, Thermo Fischer Scientific), in PBS-Tw at RT for 1 h. Following three further washes for 10 min each in PBS-Tw, the membranes were detected with the appropriate laser using a Typhoon FLA 9500 scanner (Amersham, UK).

#### Fibril digestion with proteinase K

Fibrils were collected at the end of the aggregation process by centrifugation (~ 16,000*×g*, 30 min) and washed once with PBS to remove soluble protein. 5 µM solutions of fibrils were incubated with increasing concentrations of proteinase K (0, 1, 2, 5 and 10 µg/ml) for 20 min at 37 °C. The samples were then separated by SDS-PAGE before transfer to a nitrocellulose membrane. The membrane was blocked with 5% Milk in PBS-Tw and incubated with a 1:1000 dilution of the 5C2 anti-αsynuclein primary antibody (Enzo Life Sciences, NY, USA) overnight at 4 °C. Following this, the membrane was incubated with a 1:2000 dilution of the 647 Alexa Fluor anti-mouse secondary antibody (Invitrogen, Thermo Fisher Scientific) before imaging using a Typhoon FLA 9500 scanner. For each condition, each band intensity was quantified using the Fiji Software and normalised data was plotted using GraphPad Prism (GraphPag Software, CA, USA).

### Enzyme-linked immunosorbent assay (ELISA)

To assess the binding of Gal3 to αSyn monomers and fibrils, a range of Gal3 concentrations (0.1–5 µM) was first immobilised onto a 96-well Maxisorp ELISA Plate (Nunc) and incubated at RT for 1 h with constant shaking at 350 rpm. The plate was then washed six times with TBS (20 mM Tris, pH 7.4, 100 mM NaCl) and blocked with 5% bovine serum albumin (BSA) in TBS. The plate was again washed six times with TBS before incubating with 2 µM αSyn monomers, 1st generation fibrils or 2nd generation fibrils at RT for 1 h with constant shaking at 350 rpm. The plate was then washed and incubated with a 1:5000 dilution of HRP conjugated anti-α-synuclein antibody (807,806, BioLegend, San Diego, CA, USA) in 5% BSA-TBS at RT for 1 h with constant shaking at 350 rpm. Finally, the plate was washed three times with TBS, twice with TBS supplemented with 0.02% Tween20 and again with TBS three times. The amount of bound αSyn was quantified using the 1-step Ultra TMB-ELISA substrate (Thermo Fisher Scientific). The absorbance at 450 nm was read using the CLARIOstar Plus plate reader (BMG Labtech, Aylesbury, UK).

### Electron microscopy

Sonicated PFFs were subjected to electron microscopy studies. A conventional protocol for negative staining was applied. First, a 5-µl drop of PFF or PFF with Gal3 for 30 min (PFFgal3) diluted in PBS solution was placed on top of a 100-mesh copper grid (Electron Microscopy Science, Hatfield, PA, USA). After incubation for 20 min at RT, the adhered sample was counterstained with 2% uranyl acetate in an aqueous solution for 15 min. Later, excess uranyl acetate was eliminated, and the sample was allowed to dry at RT. Images were obtained in ZEISS Libra 120 electron microscope (Zeiss, Oberkochen, Germany).

### Infrared spectroscopy

10 µM αSyn PFF was incubated in the absence or presence of 20 µM Gal3 in PBS in quiescence overnight at 37 ℃. After incubation, the insoluble protein fraction was separated by centrifugation at maximum speed using a benchtop centrifuge and washed by resuspending the pellet in PBS (900 ml of either PBS added to each tube for washing and centrifuged at maximum speed (> 10,000×*g* for 15 min). Volume is retired with pipet tips. This step is repeated twice.

After the second washing step, the pellet was resuspended in 100 µL of MiliQ water and 1 µL of suspension was deposited on the CaF_2_ window and dried under airflow. Infrared spectroscopic measurements were performed using photothermal optical infrared microspectroscopy at the SMIS beamline of the SOLEIL synchrotron (France). 1 µl of simple was deposited on the CaF_2_ window and dried under airflow. The photothermal effect was detected through the modulation of the CW 532 nm laser intensity induced by an infrared (IR) laser. The IR source was a pulsed, tuneable quantum cascade laser, scanning from 1800 to 1500 cm^−1^ at an 80-kHz repetition rate. Further details about the fundamentals of the technique and the instrument itself can be found in previous references [48, 65].

Spectra were averaged for 10 to 20 scans with 1 s acquisition time per spectra to generate data of sufficient signal-to-noise ratio. IR power was set at 22% to avoid photodamage. The probe power was set to 6%; aluminized mylar background standard was used as a background. Optical photo thermal infrared microspectroscopy (OPTIR) spectra were normalized to the maximum intensity. For peak separation and to avoid sample thickness and background contribution, second-order derivatives using the Savitsky − Golay algorithm with a 10-point filter with 3rd polynomial order were caclulated to increase the number of discriminative features [16].

### Cell lines and primary cultures

Rat dopaminergic N27 cells (RRID:CVCL_D584) were used for in vitro assays. Cells were cultured in 96-well plates, 5000 cells/well and maintained in RPMI 1640 media (Gibco, Thermo Fisher Scientific) supplemented with 1% penicillin–streptomycin (Sigma-Aldrich) and 10% Fetal Bovine Serum (FBS) (Gibco, Thermo Fisher Scientific). 24 h after plating, media was replaced, FBS concentration was kept at 2.5%, and cells were treated with 1.5 µM of PFF and/or 3 µM of Gal3 for 48 h. Cells were fixed with 4% PFA for 15 min.

Primary neuronal cultures were established following the ethical guidelines and approved by the Lund University Ethical committee (M46-16). Primary neurons were isolated from WT mouse embryos on embryonic day 16, as described before [40], 2000 cells per well were seeded on a 96-well plate pre-coated with poly-d-lysine (Sigma Aldrich) and then rinsed in autoclaved distilled water. Cell suspensions were plated in Dulbecco’s modified Eagle medium (DMEM) (Thermo Fisher Scientific) containing 10% FBS and 1% penicillin–streptomycin; after 3–5 h, media were exchanged for FBS-free complete Neurobasal medium (Gibco, Thermo Fisher Scientific). Primary neuronal cultures were maintained in a Neurobasal medium supplemented with glutamine, B27 (Gibco, Thermo Fisher Scientific), and penicillin–streptomycin and treated with 1 µM of Gal3 and 0.5 µM of PFF on the 7^th^ day in vitro. 10 days later, neurons were fixed in 4% PFA for 15 min. All the experiments were repeated 3–4 times; one embryo corresponded to one set of cultures.

### Immunofluorescence

Six months after the intranigral injection, mice were sacrificed and intracardially perfused with 0.9% NaCl for 4 min followed by 4% PFA for 4 min. Fixed brains were collected, cryoprotected in 30% sucrose, and later frozen in cold isopentane (− 40 ℃). After freezing, a cryostat was employed to cut samples at 30 µm thickness in the coronal plane. Sections were collected serially and quickly submerged in an anti-freezing buffer composed of 30% glycerine (Sigma Aldrich) and 30% ethylene glycol (Sigma Aldrich) diluted in phosphate buffer.

For immunofluorescence staining, free-floating sections were first washed from the anti-freezing solution with PBS and later treated with citrate buffer (Nzytech) for antigen retrieval by incubating the sections at 80 ℃ for 30 min. Secondly, sections were rinsed at RT for 1–2 h in PBS with the addition of 1% Triton-X100 (PBS-T) for cell permeabilization and 5% Bovine Serum Albumin (BSA) (Sigma Aldrich) for tissue blockade. Once sections were permeabilized, primary antibody recognition was let to occur overnight at 4 ℃ (Table 1). The following day, the unbound primary antibody was removed adequately with sequential PBS washes. After that, 1 h incubation with appropriate conjugated secondary antibodies (Donkey Alexa Fluor™ 488, 546 and 647) from Invitrogen™ (Thermo Fischer Scientific) diluted 1:300 in PBS was carried out. Sections were finally washed and mounted on a clean slide. A drop of ProLong Gold Antifade Mountant (Invitrogen, Thermo Fischer Scientific) was applied to preserve tissue fluorescence and seal the coverslip to the slide.

**Table 1:** Antibodies used for immunostainings

Antibody	Company	Headquarters	Reference	Dilution
Sheep anti-TH	NOVUS Biologicals, Bio-techne	Minneapolis, MN, USA	NB300-110	1:500
Rabbit anti-hSYN	Abcam	Cambridge, UK	AB138501	1:1000
Goat anti-GAL3	R&D Systems, Bio-techne	Minneapolis, MN, USA	AF-1197	1:500
Rabbit anti-αSYN	Cell signaling Technologies	Danvers, MA, USA	D37A6	1:1000
Mouse anti-NEUN	Millipore, Merck	Darmstadt, Germany	MAB377	1:200
Rat anti-GAL3, clone M3/38	Millipore, Merck	Darmstadt, Germany	MABT51	1:500
Rabbit anti-pSYN P129	Abcam	Cambridge, UK	ab168381	1:500
Rat anti-LAMP1	DSHB	Iowa City, IO, USA	H4A3	1:50
Rabbit anti-LC3B	Cell signaling Technologies	Danvers, MA, USA	2775	1:500
Mouse anti-VDAC1	Abcam	Cambridge, UK	ab14734	1:500
Rat anti-CD11B	AbD serotec (Bio-Rad)	Hercules, CA, USA	MCA74G	1:200
Chicken anti-MAP2	Abcam	Cambridge, UK	ab92434	1:250

Confocal fluorescent images were obtained from ZEISS LSM 7 DUO (Zeiss). All images were captured under the same conditions for unbiased comparison.

#### Stereological cell counting

Single immunofluorescent staining for Tyrosine Hydroxylase (TH) was employed to effectively count the total number of dopaminergic neurons in the SN. A total of 6–8 serial slices containing the mesencephalon were used.

Samples were placed under an OLYMPUS BX61 (Olympus, Tokyo, Japan) epifluorescence microscope. SN *pars compacta* was delimited by experimenter expertise and with the help of a mouse anatomical map. The stereological analysis was conducted using newCAST software (Visiopharm, Hørsholm, Denmark), specially designed for unbiased stereological studies.

Delimited volume was calculated with the software using the Cavalieri Points approach, and a 63 × immersion oil objective was employed for precise cell counting. The software unbiasedly selected microscope positions. At least 200 cells were counted for each SN following stereology guidelines, and the estimated total number was later calculated based on counted neurons, counted volume and total estimated volume.

In the case of our study, because of ipsilateral damage, data is presented as fold to the non-injected side to decrease deviation due to individual variability.

#### Dendritic tree measure

Single immunofluorescent staining for TH was employed to measure dopaminergic arborisation. A total of 6–8 serial slices containing the mesencephalon were used. Whole SN images were taken in OLYMPUS BX61 (Olympus) epifluorescence microscope. SN *pars reticulata* (SNpr) was delimited by experimenter expertise and the help of a mouse anatomical map. ImageJ^®^ software was used for the TH area measure. In the case of our study, because of ipsilateral damage, data are presented as fold to the non-injected side to decrease deviation due to individual variability.

#### LB-like counting

Double immunofluorescence for TH and hSYN was used to detect the percentage of neurons that present hSYN deposits staining. 6–8 serial slices were also used for LB-like counting.

Confocal images obtained from ZEISS LSM 7 DUO were employed. SNpc was delimited based on TH staining using ImageJ software. The area was calculated, and an unbiased counting position was selected. At least 200 neurons were counted for each animal. Rounded intense accumulation of hSYN was determined as positive by the experimenter. Data are expressed as a percentage of positive accumulation per total dopaminergic neurons counted.

#### Human immunofluorescence

Dementia with LB (DLB) samples were provided by Skåne University Hospital (Lund) and processed at Lund University, while PD samples were supplied by Hospital Universitario Virgen del Rocío and Biobanc-Hospital Clínic-IDIBAPS, and were processed at the University of Seville. The regional ethical review board in Sweden (06,582–2019) and Spain (0422-N-17 and HCB/2020/1285) approved the study. *Post-mortem* samples from DLB and PD patients were received as paraffin-embedded samples. Thus, tissue was first deparaffinised with xylene for 10 min, followed by rehydration with decreasing ethanol concentrations: 100%, 90%, 70% and 50% until the final distilled water step.

Antigen retrieval was applied to the samples to improve antibody linking. Samples were incubated in a 0.1 M sodium citrate solution and heated in a microwave for 5 min. After repeating the previous step, samples were let to recover RT for 20 min. A blocking step consisting of sample incubation in PBS-T 0.05% and 10% BSA was included to improve the signal.

Incubation with primary antibody (Table 1) was made overnight in a humidity chamber at 37 ℃ diluting it in PBS-T 0.05% and 5% BSA until desired concentration. After six washing steps with PBS, we proceeded with secondary antibody (Invitrogen, Thermo Fischer Scientific) incubation for 1 h at RT. For LB visualisation, samples were incubated with Methoxy-X04 100 µM for 10 min (Tocris Bioscience, Bristol, UK). After that, samples were rinsed three times in PBS; later, samples were immersed in 70% ethanol for 5 min, and autofluorescence was quenched with Autofluorescence Eliminator Reagent (Millipore, Merck) for 5 min; sections were then rinsed in 70% ethanol for three times and 50% glycerol was applied as mounting media, coverslip placed and sealed.

Images were obtained from ZEISS LSM 7 DUO confocal microscope and Nikon A1RHD Confocal (Nikon, Tokyo, Japan). High-resolution images were obtained by attaching the module DeepSIM (Crestoptics, Rome, Italy) to a Nikon A1RHD Confocal.

The presence of GAL3 in different hSYN deposits was evaluated through confocal microscopy with the Leica Stellaris 8 Falcon. First, the entire section was scanned using a 10X objective to detect hSYN strains. Next, the strains were classified using a 63X oil objective. Classification was made based on Methoxy-X04 positivity to discriminate between Pale Bodies (Methoxy-X04^−^) and LB (Methoxy-X04^+^), the number of Methoxy-X04^+^ cores was used to discriminate single and multiple LB. GAL3 presence was also determined. Quantification and stratification was made by a blind researcher.

#### Immunohistochemistry of human sections

Immunohistochemistry detection of GAL3 in human samples was performed similarly to the immunofluorescence protocol. Samples were deparaffinised, and antigen retrieval was performed as previously explained. After antigen retrieval, samples were washed three times in PBS and later immersed in a solution of 99% methanol and 1% H_2_O_2_ for 15 min. The blocking step, primary antibody incubation (Rat anti-GAL3, Millipore) and secondary antibody incubation (anti-rat biotinylated antibody, Invitrogen) was performed as previously explained. After secondary antibody washes, samples were incubated for 1 h at RT with a mix of reagents A and B from VECTASTAIN® Elite® ABC-HRP Kit (Vector laboratories, Newark, CA, USA) as indicated by the manufacturer. Samples were then washed six times with PBS before incubation with DAB Substrate Kit (Vector laboratories) for approximately 5 min. Finally, samples were repeatedly washed with PBS, 50% glycerol in PBS was applied as mounting media, and the coverslip was placed and sealed.

Images were obtained from an Olympus VS-120 slide scanner microscope.

#### Immunofluorescence of cell cultures

A direct immunofluorescence procedure was done in the multiwell plates to visualise primary and cell line cultures. Cells were first washed three times in PBS and later blocked with 5% BSA and 0.1% Triton in PBS for 1 h under gentle agitation. Then, cells were incubated with primary antibody diluted in PBS-T 0.1% at RT overnight (Table 1). After repeated washes, cultures were incubated with secondary antibodies diluted in PBS for 1 h at RT under gentle agitation. Finally, cell nuclei were counterstained with Hoechst (Sigma Aldrich) at 1:5000 dilution in PBS. Plates were visualised under a 720 LSM Zeiss DUO confocal microscope, and cells were counted using Operetta^®^ CLS High Content Analysis System (PerkinElmer, Waltham, MA, USA).

### Protein analysis

For protein analysis, mice brains were carefully dissected to obtain fresh striatum and mesencephalon quickly frozen in liquid nitrogen (for more information, see the *Treatment and Sacrifice* section). All the following procedures were carried out in the Experimental Neuroinflammation Laboratory (BMC B11) at Lund University (Sweden).

#### Protein extraction and quantification

Protein extraction was the first step before any additional procedure. Initially, samples were embedded in cold RIPA buffer (Sigma Aldrich) supplemented with proteinase and phosphatase inhibitor (Roche, Basel, Switzerland) and later sonicated for 10 s for membrane disruption. Samples were then centrifuged for 10 min at 10,000 rpm to isolate the total proteins that remained in the supernatant.

Protein concentration was calculated using Pierce™ BCA Protein Assay Kit (Thermo Fischer Scientific). As recommended by the manufacturer, BSA standard dilutions were carefully made in RIPA buffer in a working range of 2000–20 µg/ml. Samples were then incubated with supplied reagent at 37 ℃ for 30 min. Later, 562 nm wavelength was measured in a Benchmark Plus microplate reader (Bio-Rad, Hercules, CA, USA). Samples were diluted to a final concentration of 2 µg/µl with RIPA buffer.

#### Western blot

Quantified proteins were first denaturalized by adding an equal volume of Laemmli buffer (Bio-Rad) supplemented with β-mercaptoethanol (Sigma Aldrich). After that, proteins were separated by SDS-PAGE using 4% pre-cast acrylamide gels (Bio-Rad) in TGS buffer (Bio-Rad). After electrophoresis, proteins were transferred from the gel to a nitrocellulose membrane (Bio-Rad) using a TransBlot turbo system from Bio-Rad. Membranes were then blocked for 1 h with 3% skim milk (Sigma Aldrich) in PBS. Membranes were subsequently washed PBS-Tw prior to overnight incubation with primary antibodies (Table 2). After several washes with PBS-Tw, the membranes were incubated with peroxidase-conjugated secondary antibody for 2 h at 1:10,000 dilution. After final washes with PBS-Tw, blots were developed using ECL Clarity and ChemiBlot XRS + system from Bio-Rad. β-actin was used as a housekeeping protein, and data are expressed as a percentage of the fold to actin.

**Table 2:** Antibodies used for western blot

Antibody	Company	Headquarters	Reference	Dilution
Rabbit anti-hSYN	Abcam	Cambridge, UK	AB138501	1:1000/1:5000
Rabbit anti-pSYN P129	Abcam	Cambridge, UK	AB168381	1:3000
Goat anti-GAL3	R&D Systems, Bio-techne	Minneapolis, MN, USA	AF-1197	1:1000
Mouse 5C2 anti-αSyn	Enzo Life Sciences	Farmingdale,NY,USA	ALX-804-656-R100	1:1000
Mouse anti-β-ACTIN-Peroxidase	Sigma Aldrich	St Louis, MO, USA	A3854	1:10,000

#### Cytokine quantification

To measure cytokine levels in SN and striatum, we took advantage of ELISA multiplex technology supplied by Meso Scale Diagnostics (Rockville, MD, USA). We selected a proinflammatory panel composed by IFNγ, IL-1β, IL-2, IL-4, IL-5, IL-6, IL-10, IL-12 and TNFα. 50 µg of total protein extract diluted in RIPA buffer from SN and STR of animals was used. Plates were incubated for 2 h at RT and constant shaking. Later, samples were rinsed three times with PBS-Tw 0.1%. Then, appropiate secondary antibodies supplied in the kit were added to the plate and incubated for 2 h at RT and constant shaking. After incubation, three PBS-Tw 0.1% washes were applied again. The plates were developed using the 2X reading buffer diluted to a factor of 1X with MiliQ water, and the plates were read using the QuickPlex Q120 reader from Meso Scale. The detection ranges of the different cytokines measured were as follows: IFN-γ (938–0.229 pg/ml), IL1β (1670–0.408 pg/ml), IL2 (2630–0.642 pg/ml), IL4 (1660–0.405 pg/ml), IL5 (967–0.236 pg/ml), IL6 (5720–1.40 pg/ml), IL10 (3410–0.833 pg/ml), IL12 (32,200–7.86 pg/ml) and TNF-α (627–0.153 pg/ml).

#### ELISA from human samples

Cortex and SN from human samples were weighed, and RIPA buffer was prepared, including Protein Inhibitor Cocktail (Thermo Fischer Scientific) to prevent protein degradation and PhosphoStop (Roche) from inhibiting the enzymatic activity of phosphatases. The tissues were then sonicated in 30 s pulses in ice to avoid protein degradation until complete disgregation. Protein concentration was measured using a Pierce™ BCA Protein Assay Kit (Thermo Fischer Scientific) following the manufacturer instruction. Human Galectin-3 ELISA Kits (Abcam) were used to measure GAL3 levels (detection range 2000–58.8 pg/ml) in tissue homogenate. 1 ng of total protein was used for the assay. The protocol was carried out according to the manufacturer instructions. A Biotek Synergy 2 was used to read both the BCA at a wavelength of 562 nm, and the ELISA GAL3 assay at the recommended wavelength of 450 nm.

### Gene expression analysis

#### RNA extraction

Serial sections from SN were used for RNA extraction and RT-PCR analysis. After mechanical disaggregation, FFPE-RNA Purification Kit (Norgen, Thorold, ON, Canada) was employed to obtain total RNA. Following manufacturer guidelines, the provided digestion buffer was first used for lysate preparation supplemented with proteinase K for reversing formalin-induced crosslink between RNA and proteins. Samples were incubated in this buffer at 55 °C for 15 min and then incubated at 80 °C for 15 min. After incubation, samples were placed on a silica-membrane spin column and centrifuged at > 3500 g for 1 min until the entire lysate volume had passed through the spin column. Next, the column was washed two times with provided washing solution and centrifuged at > 3500 g for 1 min. Finally, RNA was eluted with nuclease-free water.

RNA quantity was assessed by measuring 260 nm wavelength on NanoDrop™ 2000 (Thermo Fischer Scientific). RNA quality was also controlled based on the 260/280 ratio which identifies protein contamination, and the 260/230 ratio which determines salts and phenol contamination.

#### RT-PCR

RNA obtained from tissue sections was then processed to obtain viable cDNA for Real Time-Polymerase Chain Reaction (RT-PCR) detection. For that purpose, RevertAid First Strand cDNA Synthesis Kit (Thermo Fischer Scientific) was used to transform up to 1 µg of RNA into the corresponding cDNA with the mixture of components given by the manufacturer: 2 µl dNTPs, 1 µl Reverse Transcriptase, 1 µl Random Primers, 1 µl RNAse inhibitor and 5 µl of the provided buffer. Samples were carefully mixed and spun down for later incubation that consisted of a single cycle of three steps: 5 min incubation at 25 ℃, 60 min incubation at 42 ℃ and a final step at 70 ℃ for 5 min.

Obtained cDNA was used directly for RT-PCR or frozen at − 20 ℃. The following primers from Sigma Aldrich were all self-designed using BLAST® software from NCBI and previously tested in our lab: *Atcb* (5′- GGCTATGCTCTCCCTCACG, 5′- CTTCTCTTTGATGTCACGCACG); *Gapdh* (5′- GTGTTTCCTCGTCCCGTAGA, 5′ AATCTCCACTTTGCCACTG); *Trem2* (5′- GTTTCTTGCAGCCAGCATCC, 5′- GGGTCCAGTGAGGATCTGAAG); *Mertk* (5′- CTGTCCAAATCCACAATGCCAC; 5′- GTTGACGAGGGTGCGTAATC); *Lgals3 (*5′- *CCCAACGCAAACAGTATCACTC;* 5′- *CCCAGTTATTGTCCTGCTTCG)*. The RT-PCR reaction was carried out by mixing our cDNA sample with SensiFAST™ SYBR No-ROX kit (Bioline) mix and the corresponding primer pair. This kit provides a single component that includes dNTPs, DNA Polymerase and buffer. PCR mix was made by adding 5 µl of SensiFAST™ SYBR No-ROX mix, 0.4 µl of each primer, and 4.2 µl of cDNA sample. RT-PCR was performed using LightCycler 480 (Roche). PCR conditions were determined by the DNA Polymerase provider. They consisted of a single incubation of 2 min at 95 ℃ for polymerase activation and 40–50 cycles of denaturation (95 ℃ for 5 s), annealing (60 ℃ for 10 s) and extension (72 ℃ for 15 s). The results were calculated using the delta Ct method and represented as fold to control group values. *Actb* and *Gadph* were used as housekeeping genes. The melting curves were used as a control point for individual reaction validation.

## Results

### Galectin-3 is present in α-synuclein strains from DLB and PD patients

Campbell and colleagues [20] have recently identified GAL3 in the outer layer of LB from PD patients. This finding can be interpreted in different ways, including GAL3 recruitment to damaged vesicles considering that the GAL3 carbohydrate recognition domain (CRD) may adhere to the glycans present on the intraluminal membrane proteins of endosomes and lysosomes [49]. Previously, we have identified a prominent role of GAL3 in amyloid β aggregation [7]; hence, the possibility that GAL3 may be involved in αSYN aggregation and LB formation is plausible. Since LB are present in a heterogeneous group of neurodegenerative diseases, we first wanted to know if the presence of GAL3 is a widespread feature of LB. For this purpose, we assessed its presence and localisation in post-mortem samples from patients earlier diagnosed with dementia with LB (DLB). Dual confocal immunofluorescence confirmed the presence of GAL3 in LB from DLB, where GAL3 was preferentially associated with the outer ring of LB but was also present in the inner parts (Supplementary Fig. 1a).

**Fig. 1:**
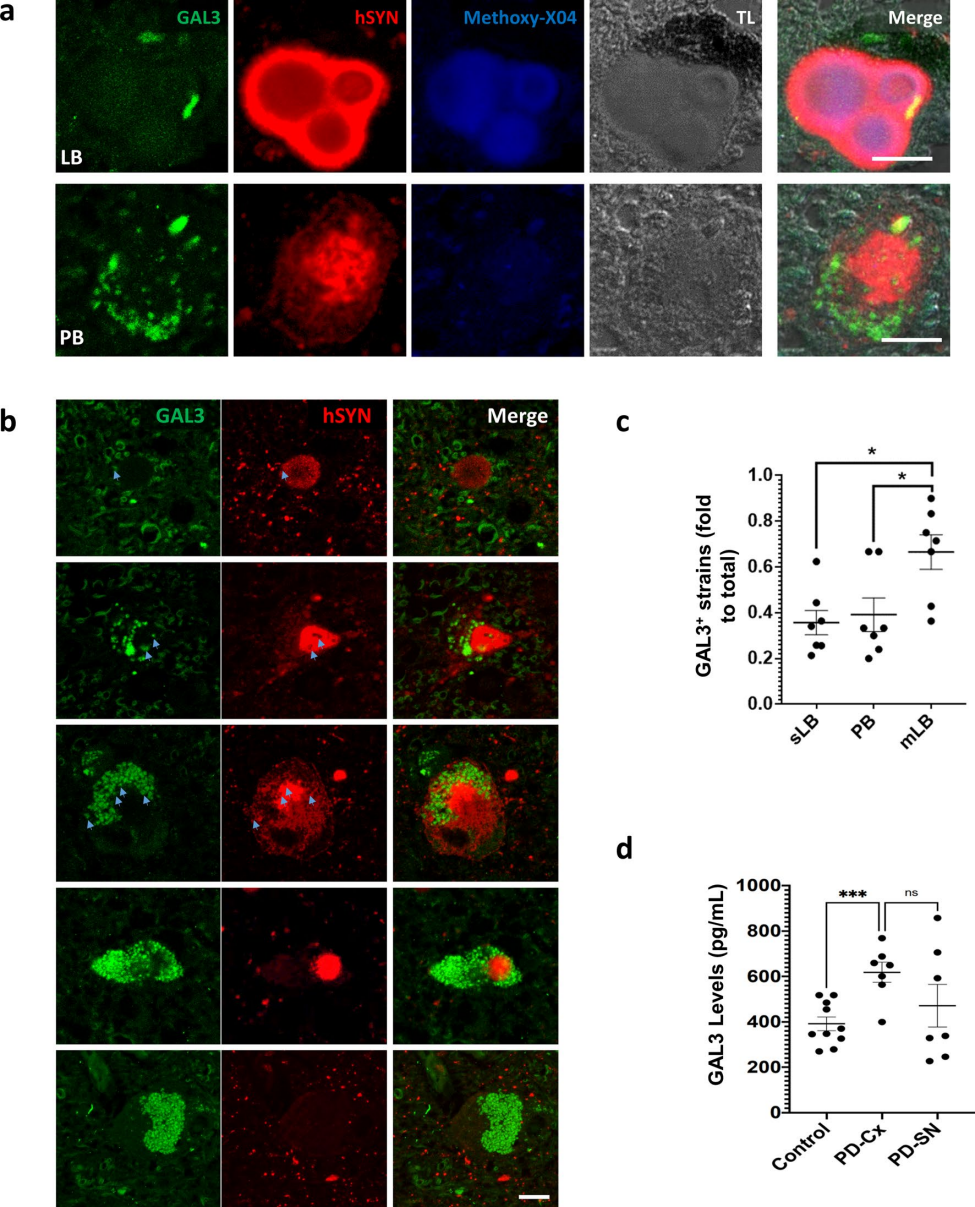
Galectin-3 (GAL3) is associated with Lewy Bodies (LB) and Pale Bodies (PB) in PD patients. **a** Immunofluorescence analysis of GAL3 in association with distinct forms of human α-synuclein (hSYN) aggregation. Β-sheet structure marker Methoxy-X04 was used to discriminate between LB and PB. Multiple core LB and PB are shown. GAL3 is present in both types of aggregates independently of neuromelanin presence. Scale bar 10 µm. **b** GAL3 is present in a diverse subset of hSYN accumulations with a precise negative correlation (blue arrows). Scale bar 10 µm. **c** Proportion of hSYN aggregates that are associated with GAL3. Methoxy-X04 was used as a specific marker of LB. Single (sLB) and multiple core LB (mLB) were discriminated (*p* < 0.05). **d** Protein levels of GAL3 measured by ELISA in the Cortex of Control and PD Patients (PD-Cx) (*p* < 0.001), and in the Substantia nigra (PD-SN) of PD patients

The analysis was also performed on brain samples from PD patients. In line with previous studies [20], GAL3 appeared to be associated with the outer layer of LB from neuromelanin-containing cells (Fig. 1a). However, we also identified GAL3 related to different types of αSYN strains in the ventral mesencephalon of PD patients exhibiting typical features of Pale Bodies (Fig. 1a lower panel). These data are important because the formation of Pale Bodies appears as critical elements preceding LB formation [19], thus raising the possibility that GAL3 drives αSYN fibril reorganisation linked to LB formation. Interestingly, when analysing Pale Bodies, we found strains showing an intense core highly positive to αSYN surrounded by more diffuse staining (Fig. 1a lower panel and b). Remarkably, we found GAL3 puncta-like staining within these αSYN deposits that poorly colocalize with αSYN staining (Fig. 1a and b). In order to determine the interaction between GAL3 and αSYN, we identified a broad subset of αSYN strains immunopositive to GAL3 and exhibiting features associated with different stages known to precede LB formation [19]. From compacted and rounded deposits to more diffused accumulations of αSYN with visible halo, GAL3 can be sourrunding but poorly colocalizing with αSYN staining (Fig. 1b). This analysis suggests a role of GAL3 in the evolution of αSYN strains from pale bodies to LB. To further investigate this association, we quantified the presence of GAL3 in different αSYN strains based on reactivity to amyloid marker Methoxy-X04. Thus, we were able to distinguish between Methoxy-X04^−^ PB, Methoxy-X04^+^ single LB, and multilobar LB. We found that GAL3 is present in about 40% of PB and single LB and is significantly increased in multilobar LB (Fig. 1c), suggesting a potential role of GAL3 in LB shape. Interestingly, LB represent the vast majority of the αSYN strains counted, although we are aware that PB may be undestimated due to their less defined structure and lower αSYN accumulation (Supplementary Fig. 1b). Previous studies have revealed increased serum levels of GAL3 in PD patients [13, 64]. We wondered if this increase also occurred in the SN of these patients. Consequently, we measured levels of GAL3 by ELISA and were able to detect GAL3 in SN from PD. Unfortunately, we did not have access to SN from age-matched controls; yet, we decided to measure the levels of GAL3 in the same PD patients' cortex and compare them to the cortex of control patients. The levels of GAL3 in the cortex of PD patients were significantly increased compared with the cortex of age-matched controls (Fig. 1d). Overall we conclude that GAL3 is present at detectable levels in SN from PD patients, which is a prerequisite for playing a significant role in the disease. Indeed, GAL3 levels were found elevated in the cortex of PD patients suggesting a potential correlation with the pathology.

**Fig. 2:**
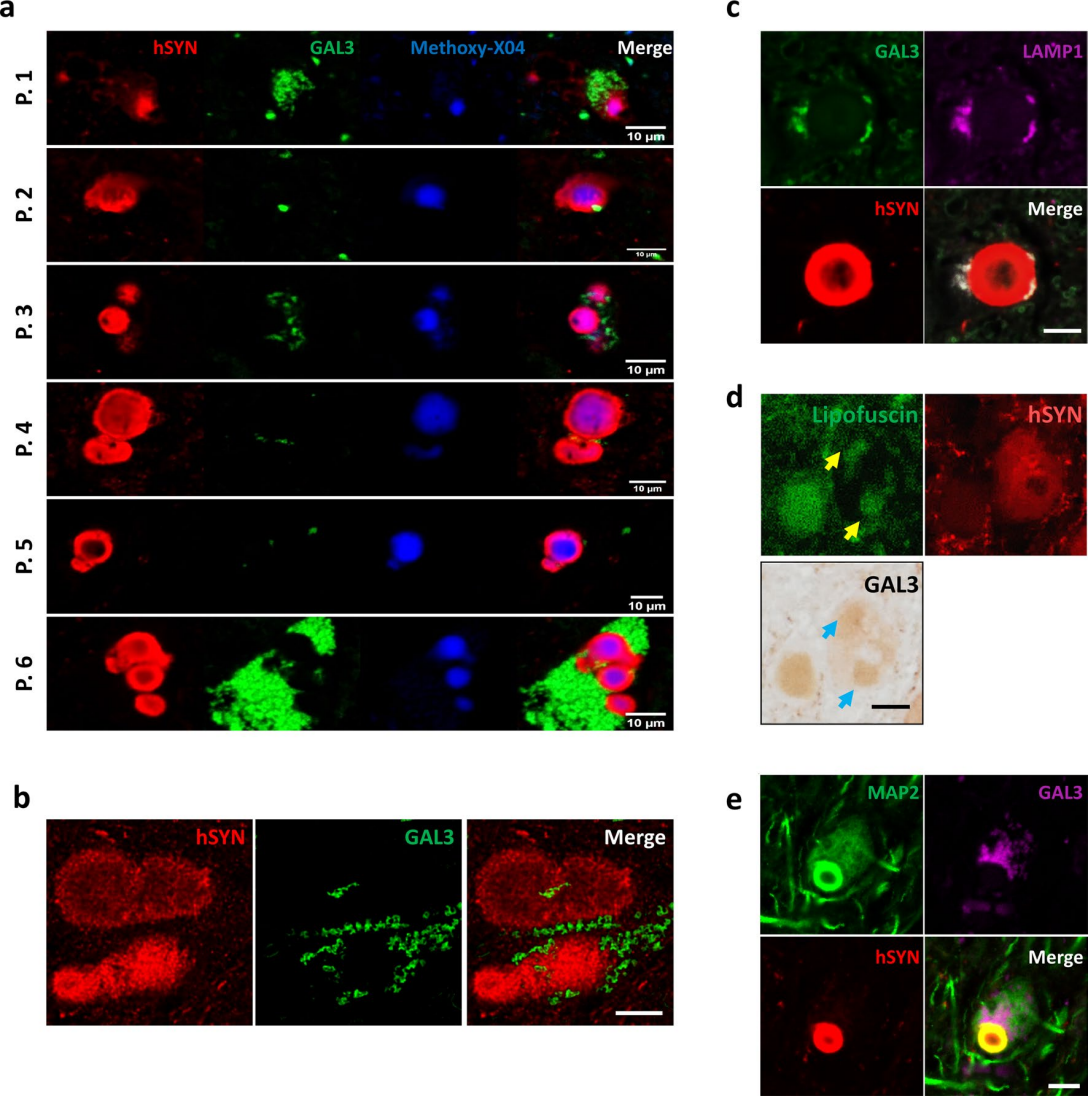
GAL3 variably associates with lysosomes in the outer layers LB in all the studied patients. **a** GAL3 surrounding LB was found in all 6 patients studied (P.1–6). Variable amount of GAL3 vesicles was found. Note lower hSYN staining in the presence of GAL3. Scale bar 10 µm. **b** High resolution microscopy showed a ring-like pattern for GAL3 without any hSYN inside. Scale bar 10 µm. **c** Immunofluorescence analysis revealed that GAL3 is associated with recruited lysosomes (LAMP1) in the vicinities of LB. Scale bar 10 µm. **d** Combination of GAL3 immunohistochemistry with immunofluorescence showed that GAL3 is associated with autofluorescent lipofuscin vesicles in PD patients. Scale bar 10 µm. **e** Immunofluorescence analysis revealed that GAL3 accumulates inside MAP2^+^ neurons in the viccinities of LB. Scale bar 10 µm

The interaction between GAL3 and αSYN was further investigated in LB from six different PD patients included in the study (Fig. 2a), confirming that GAL3 was present in the vicinities of LB correlating with aparent alterations of the classical rounded shape. Moreover, we performed high-resolution microscopy and could confirm that no αSYN staining appeared inside GAL3 vesicles (Fig. 2b), implying that GAL3-αSYN interaction could occur at the membrane of the damaged vesicles. Interestingly, the presence of GAL3 colocalised with lysosomal and endosomal markers LAMP1 and Rab7, but not with exosomal marker CD63 (Fig. 2c and Supplementary Fig. 1c). GAL3 is known to bind the lumen of damaged lysosomes due to its affinity to the glycoproteins present in the inner part of lysosome membranes that are exposed in case of membrane rupture [1]. Additionally, we discarded any autofluorescent artifacts by applying a combination of immunohistochemistry with immunofluorescence to demonstrate that GAL3 was also present in endogenous autofluorescent lipofuscin vesicles (Fig. 2d). Supporting a pathological role of GAL3, we detected GAL3 inside MAP2 positive neurons (Fig. 2e and Supplementary Fig. 1d), but were unable to detect neither GAL3 in neuromelanin-positive cells from control patients nor colocalisation of GAL3 with other markers, including mitochondrial VDAC1 and autophagosomal LC3B (Supplementary Fig. 1e-f). Overall, these findings strongly support the view that GAL3 is actively involved in the homeostasis of αSYN strains.

### Galectin-3 prevents α-synuclein aggregation and interacts with α-synuclein fibrils in vitro

Having established the strong association between GAL3 staining and pathological αSYN strains, we next wondered if GAL3 plays a role in αSYN aggregation/disaggregation. To investigate this, we performed in vitro assays, where we monitored the effects of Gal3 on (1) the kinetic of spatial propagation/elongation of αSyn strains, and (2) the stability and solubility of these strains.

**Fig. 3:**
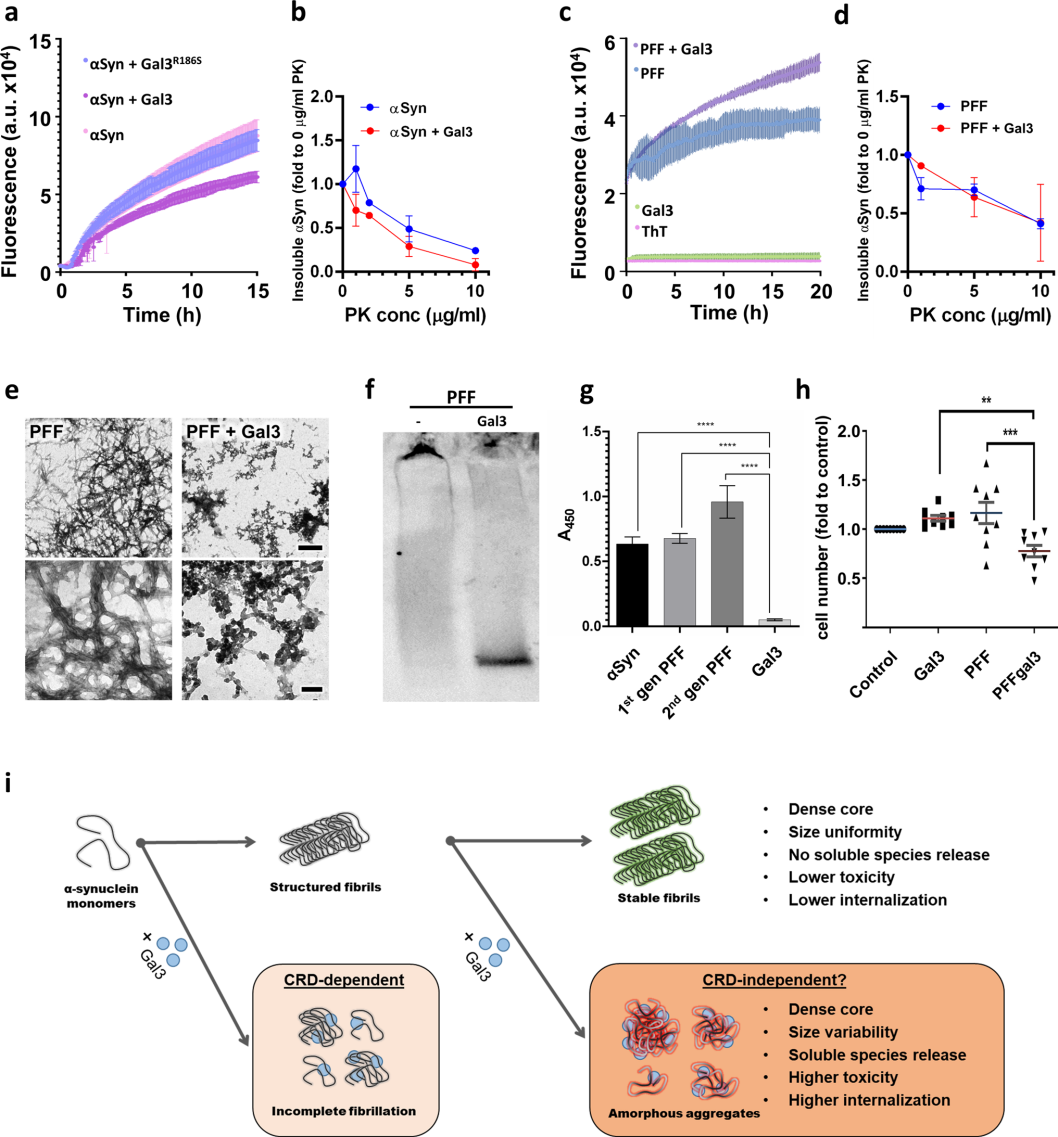
Recombinant galectin-3 (Gal3) impairs synuclein aggregation in vitro. **a** Thioflavin-T (ThT) aggregation assay showed a rapid aggregation for recombinant human α-synuclein (αSyn) that was impaired in the presence of recombinant Gal3 (purple line). Notably, carbohydrate recognition domain (CRD) mutation (Gal3^R186S^) reverted this effect. **b** Proteinase K (PK) digestion at increasing concentration of resultant conditions from a) showed a lower stability in the presence of Gal3 (red line). **c** When Gal3 was added to αSyn pre-formed fibrils (PFF) after aggregation was completed, an increased signal was observed in the presence of ThT after 15 h. **d** PK digestion at increasing concentration of resultant fibrils from (**c**) showed similar stability of PFF in the presence of Gal3. **e** Electron microscopy images after uranyl negative staining of PFF after 24 h incubation with Gal3 (right panels). Note a marked disorganization of the fibrils network after Gal3 incubation with increased shortened species (upper right panel), and the change of morphology (lower right panel) with rounded structures attached to the fibrils. Scale bar 1 µm (upper panels) and 200 nm (lower panels). **f** Native PAGE Western Blot of the final results obtained in **c**) Note that Gal3 promoted an increase in smaller soluble species released by αSyn fibrils. **g** Direct interaction of Gal3 with different αSyn species was investigated by ELISA. 2 µM Gal3 concentration were precoated in a 96 well plate and 2 µM αSyn species were incubated. 450 nm absorbance was measured to detect bounded protein. All types of species presented high affinity for Gal3 coated well compared with the control condition in absence of αSyn (*p* < 0.001). No relevant absorbance was detected in the absence of precoated Gal3 (data not shown). **h** Addition of sonicated PFF pre-incubated with gal3 (PFFgal3) for 30 min to dopaminergic cell line N27 for 48 h led to a decreased number of cells compared with PFF alone (**p < 0.01; ***p < 0.001). **i** Graphical abstract representing the hypothesis proposed based on our in vitro studies about Gal3-αSyn interaction. Gal3 could impact αSyn elongation in de novo formation of fibrils while also affecting structured fibrils with little impact on the dense core but release of small species

Seeded Thioflavin-T (ThT) assays were performed to analyse the effect of Gal3 in αSyn aggregation. As expected, αSyn in the absence of Gal3 displayed classical aggregation kinetics, as it quickly aggregated until saturation (Fig. 3a). Exogenous Gal3 significantly prevented αSyn fibrillation (Fig. 3a). Interestingly, this effect strongly depended on the carbohydrate recognition domain (CRD), as demonstrated by using the Gal3 R186S mutant protein, which lacks CRD functionality. The mutation of the CRD reversed the effect seen with the non-mutated form of Gal3, and αSyn fibrillation was like control conditions (Fig. 3a). Thus, we assessed the stability of α-synuclein fibrils formed in the absence and presence of Gal3 at the end of aggregation. To do so, we performed a proteinase K digestion assay. We collected the fibrils at the end of aggregation by centrifugation and washed the pellet to remove any soluble protein. 5 µM αSyn fibrils formed in the presence and absence of Gal3 were then incubated with increasing concentrations of proteinase K (0-10 µg/ml) for 20 min. The digested samples were then analysed by SDS-PAGE and western blotting using an antibody against the NAC region (61–95) to specifically monitor the stability of the core of the fibrils. The densitometry analysis of the western blots showed that the core of fibrils formed in the presence of Gal3 are less stable than those formed in the absence of Gal3 (Fig. 3b). Strikingly, the effect of Gal3 on αSyn aggregation is dependent on the CRD full functionality, as assessed by ThT assay (Fig. 3a and Fig. 3i), but is not dependent on the presence of glycan in the αSyn molecule. This was assessed by electrospray liquid chromatography (ES-LC) mass spectroscopy where we clearly identified a peak of 14,461 Daltons which is the expected mass of the unmodified protein (Supplementary Fig. 2a).

Next, we wondered if Gal3 could have a similar effect on 2nd generation preformed fibrils (PFF). To this end, we performed a ThT assay that revealed that, in the presence of Gal3, the fluorescence of PFFs was similar to the control (Fig. 3c and Supplementary Fig. 2b). Additionally, the fluorescence of 1-aniline-8-naphthalene sulfonate (ANS) to monitor exposed hydrophobic regions [46] was also unchanged (Supplementary Fig. 2c and d), suggesting that, overall, Gal3 does not produce major structural changes of the core of the αSyn fibrils. Interestingly, we did not observe a significant difference in the stability of the core of the fibrils by PK digestion (Fig. 3d). Next, we performed electron microscopy (EM) to investigate the morphology of PFFs exposed to Gal3. EM micrographs demonstrated that in the absence of Gal3, PFF appeared as an intricate network composed of long and thin fibrils (Fig. 3e). In contrast, PFF incubated with recombinant Gal3 appeared as a disordered accumulation of fibrils with irregular granulated morphology of variable size, including short species, known to be more reactive because of the higher proportion of fibrillary ends at the same mass concentration [12]. In this line, we also found that the PFFs have a higher tendency to release soluble protein as assessed by Native PAGE (Fig. 3f and Supplementary Fig. 2e). To obtain a more quantitative understanding of the fibrillar structures, we used optical photo thermal infrared microspectroscopy (OPTIR) [34] using the same sample preparation than EM. Our analysis showed structural changes based on the intensity differences between spectra collected from pure αSyn PFFs and PFFs incubated with Gal3 for 24 h. Analyzing OPTIR spectra, we documented the following two phenomena: (i) Gal3-specific bands disappear (1714, 1670, 1652 cm^−1^) in PFF + Gal3 spectra (Supplementary Fig. 2f, g), which we interpret as structural changes of Gal3. (ii) We observed a relative decrease in the intensity of 1634 cm^−1^ band (β-sheets) in PFF + Gal3 fibrils compared to the 1654 cm^−1^ intensity (α-structures) accompanied by a significant shift of 1668 cm^−1^ band to 1656 cm^−1^ (Supplementary Fig. 2 g, h). Since all unbound Gal3 monomers were removed during washing steps, and the concentration of αSyn used for all sample preparation was exactly the same, intensity changes in OPTIR spectra may indicate a direct interaction between Gal3 and αSyn fibrils. To validate OPTIR results, we used the same simple preparation for electron microscopy. Both EM and OPTIR results indicate a direct interaction between Gal3 and PFF. Thus, we investigated this interaction by ELISA. We coated the wells of the ELISA plate with increasing concentrations of Gal-3 followed by the same concentration of αSyn monomers, 1st generation or 2nd generation PFFs. We found that Gal3 is capable of binding to both monomers and fibrils to a similar extent (Fig. 3g and Supplementary Fig. 2i).

Interestingly, our data suggest that the disorganization effect of Gal3 on PFFs could be in a CRD-independent manner, as R186S and CRD-only Gal3 variants produced a similar release of soluble species than full-lenght Gal3, as observed by Native PAGE. In contrast, the effect of Gal3 in the inhibition of αSyn fibrillation/elongation was dependant on CRD full functionality (Fig. 3i and Supplementary Fig. 2d); however, this should be further confirmed.

A recent study by Emin and colleagues [18] demonstrated that small αSyn strains rather than larger aggregates (over 200 nm in length) are toxic and cause inflammation and permeabilisation of intracellular membranes. They discovered that smaller strains are more common in PD patients, while larger ones are in control patients. Thus, we explored the potentially toxic effects of these species in a neuronal cell line. We first sonicated αSyn fibrils to obtain shorter species that could be efficiently internalised; later, we incubated these species with Gal3 for 30 min (PFFgal3) before adding them to the cells for 48 h. We decided to sonicate the fibrils before Gal3 incubation due to the different efficiency of sonication in PFFgal3 fibrils compared with pure ones (Supplementary Fig. 2j). Interestingly, the total number of cells decreased after the addition of co-incubated PFFgal3 (Fig. 3g). The latest suggests that PFFgal3 species could be more toxic for the cells than PFF alone. This finding may have important implications in PD pathology, given the strong ability of Gal3 to interact with αSyn strains that may ultimately drive toxicity. Furthermore, we found that PFFgal3 species triggered a higher number of deposits in the cells than non-incubated PFF (Supplementary Fig. 2 k), suggesting a more efficient internalisation of PFFgal3 species, probably due increased number of small-size strains that are known to be better internalised [51].

The findings strongly indicate that Gal3 has the ability to alter the structure of αSyn PFFs. This is achieved by releasing soluble assemblies and/or monomers from the fibril ends, which results in only a moderate change in the β-sheet content of the fibrils. However, this relatively small structural change translates into a significantly higher level of neurotoxicity, which could have important implications for PD pathology.

### AAV5-hSYN nigral injection as a model for PD study

So far, the neuroprotective role(s) of GAL3 in PD has not been elucidated in any in vivo model. Hence, we evaluated the effect of GAL3 deletion in an established adenovirus-based PD mouse model overexpressing human αSYN (hSYN) or green fluorescent protein (GFP) as a control vector injected unilaterally in the SN pars compacta. Behavioural tests were performed 8 weeks and 6 months after the injection to determine progressive motor impairment. Mice were sacrificed after the last behavioural test.

**Fig. 4:**
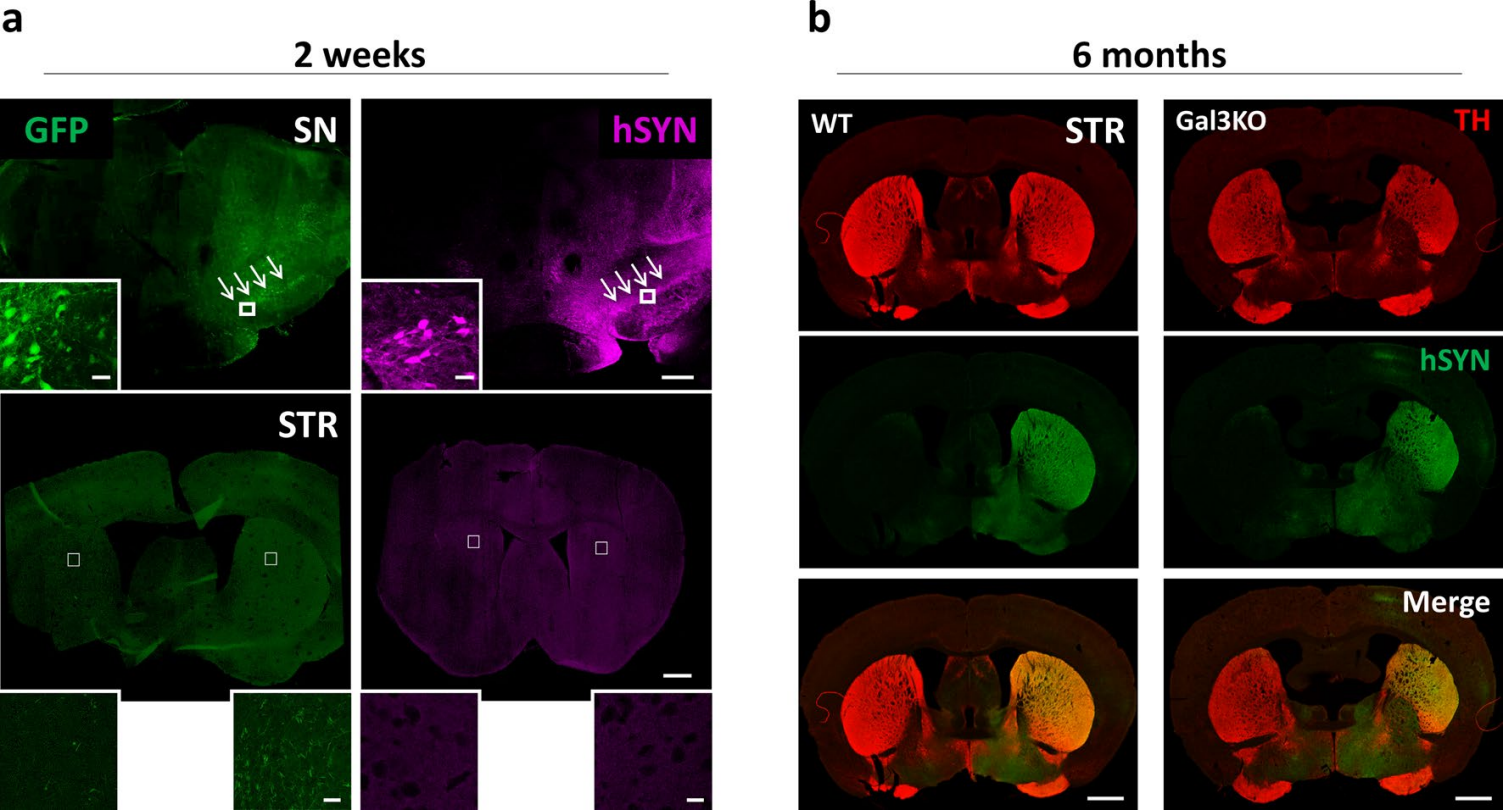
Model validation. Intranigral injection of AAV5-GFP and AAV5-hSYN. **a** Ipsilateral expression of adenovirus-associated human proteins (GFP and hSYN) in Substantia Nigra (SN) and Striatum (STR) 2 weeks after injection. Mice were injected with adenovirus in the left mesencephalon. Immunostaining of WT mice 2 weeks after injection revealed a clear ipsilateral expression in SN for both proteins but not in the STR. Scale bar 100 µm. Scale bar of amplified images 20 µm. **b** Expression of synuclein in the ipsilateral STR was evident 6 months after the injection for both genotypes indicating effective anterograde transport of hSYN. No evidence of degeneration. Scale bar 100 µm

We first determined the efficacy of our in vivo model to express hSYN within dopaminergic neurons in the SN along with its anterograde transport to the striatum. Expression of hSYN and control GFP proteins were robustly detected in the ventral mesencephalon with marked expression in the majority of nigral dopaminergic neurons 2 weeks after injection (Fig. 4 and Supplementary Fig. 3a). At this postinjection time, no significant expression of either hSYN or GFP was found in the striatum (Fig. 4a). Long-term validation was performed 6 months after injection prior to experimental analysis. Robust expression of hSYN and GFP proteins in both SN and striatum was observed with no difference between genotypes observed by immunohistochemistry or western blot (Fig. 4b and Supplementary Fig. 3b).

### Galectin-3 deletion promotes non-toxic α-synuclein strains after adenoviral injection

**Fig. 5:**
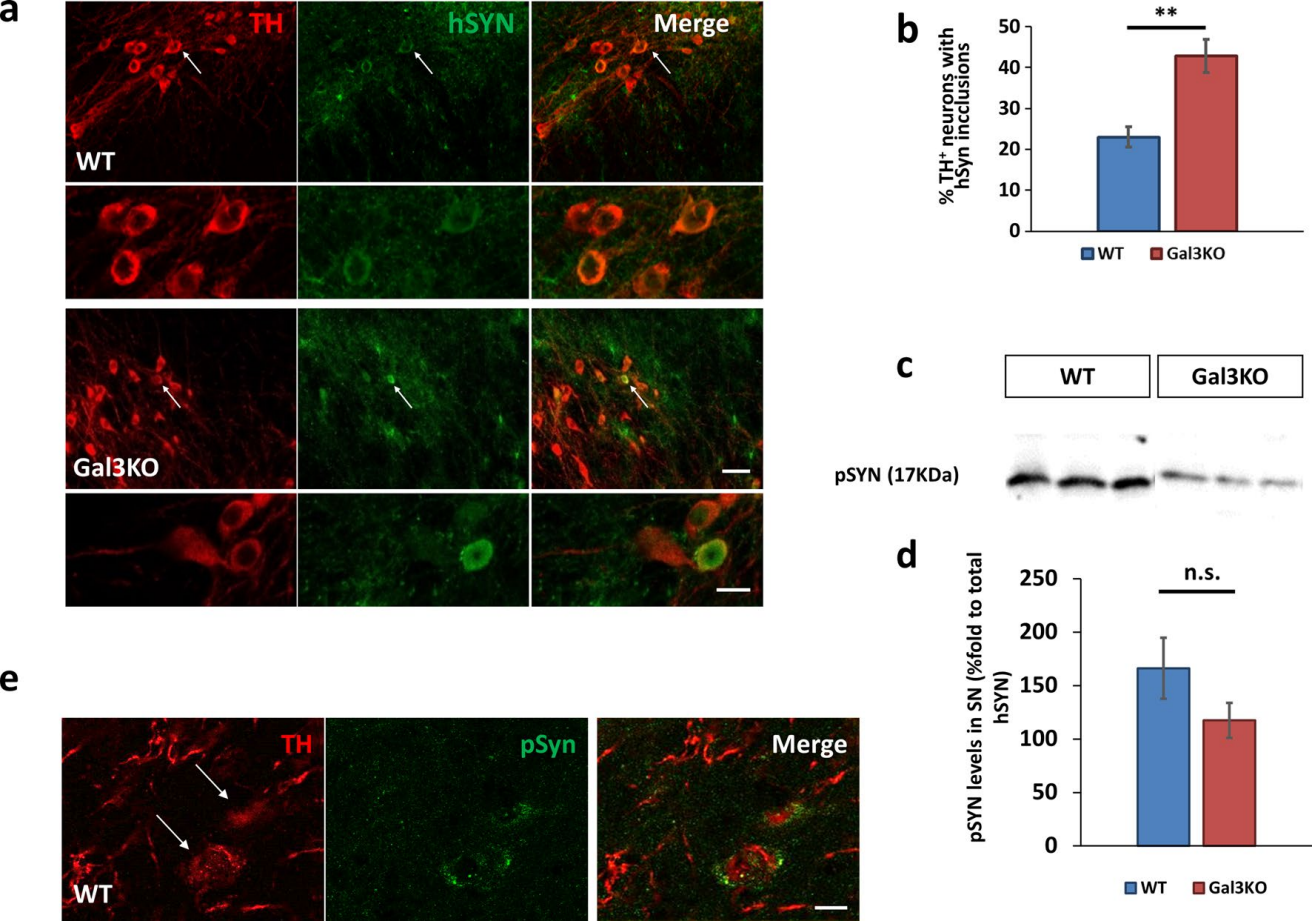
Gal3KO mice showed increased hSYN accumulation but similar phosphorylation. **a** TH/hSYN immunostaining revealed a different expression pattern for WT and Gal3KO mice. Note intracellular rounded accumulations present in Gal3KO but not in WT (white arrows and amplification below). Scale bar 50 µm. **b** TH + /hSYN + double positive stereological counting of the SNpc (*n* = 5). hSYN intracellular inclusions are present predominantly in Galectin-3 knockout mice. Data are expressed as fold to total TH + neurons (*p* < 0.01). **c** Western Blot of total Ser129 phosphorylated form of αSyn (pSYN). **d** TH/pSYN double immunofluorescence revealed unusual dopaminergic debris with presence of pSYN in WT mice, associated with an unfinished neurodegenerative process. Scale bar 5 µm. **e** Western Blot quantification, pSYN is not significantly changed in WT injected mesencephalon compared to Gal3KO. Data are expressed as percentage fold to total overexpressed hSYN (*p* = 0.059)

To investigate the state of the overexpression of hSYN in our model, we performed double immunofluorescence against hSYN and TH. We discovered a remarkably different pattern in the expression of hSYN within the SN of both animals (Fig. 5a). AAV-injected WT animals presented a classical puncta pattern corresponding to localization in synaptic terminals and mild soma reactivity with diffuse intracytoplasmic accumulation. Remarkably, Gal3KO mice overexpressing hSYN showed strong rounded intracytoplasmic inclusions of hSYN in nigral dopaminergic cell bodies reminiscent of Lewy bodies. Intracytoplasmic accumulation of hSYN was later quantified following stereological criteria (Fig. 5b). The quantification revealed a remarkable double increase of hSYN accumulation in the dopaminergic neurons in the SN of Gal3KO mice (42.83% ± 4.10 of the dopaminergic neurons in the SN of Gal3KO mice presented this type of accumulation and 23.01% ± 2.45 in WT mice). These results are in line with our in vitro experimental data demonstrating an important role of GAL3 in preventing αSYN aggregation. As previously stated, we have shown the ability of GAL3 to interact with αSYN strains leading to more toxic species. Consequently, we next wondered if the increase in hSYN accumulation observed in Gal3KO mice was also linked with increased phosphorylation of αSYN (pSYN). We first measured the total amount of pSYN in protein extracts from the ventral mesencephalon of WT and Gal3KO mice (Fig. 5c). Interestingly, we found non significant increased levels of pSYN in WT compared to Gal3KO when normalized by total hSYN (Fig. 5d), thus discarding a significant correlation between soluble levels of pSYN and hSYN accumulation in the SN. However, our data is suggestive of higher levels of soluble toxic αSYN species in WT mice compared to Gal3KO mice. In addition, immunofluorescence analysis revealed that the presence of pSYN in WT mice was inherently associated with unhealthy dopaminergic neurons displaying lower TH immunoreactivity and/or a reduction in their dendritic processes (Fig. 5e).

### Microglia-derived GAL3 is internalized by neurons

**Fig. 6:**
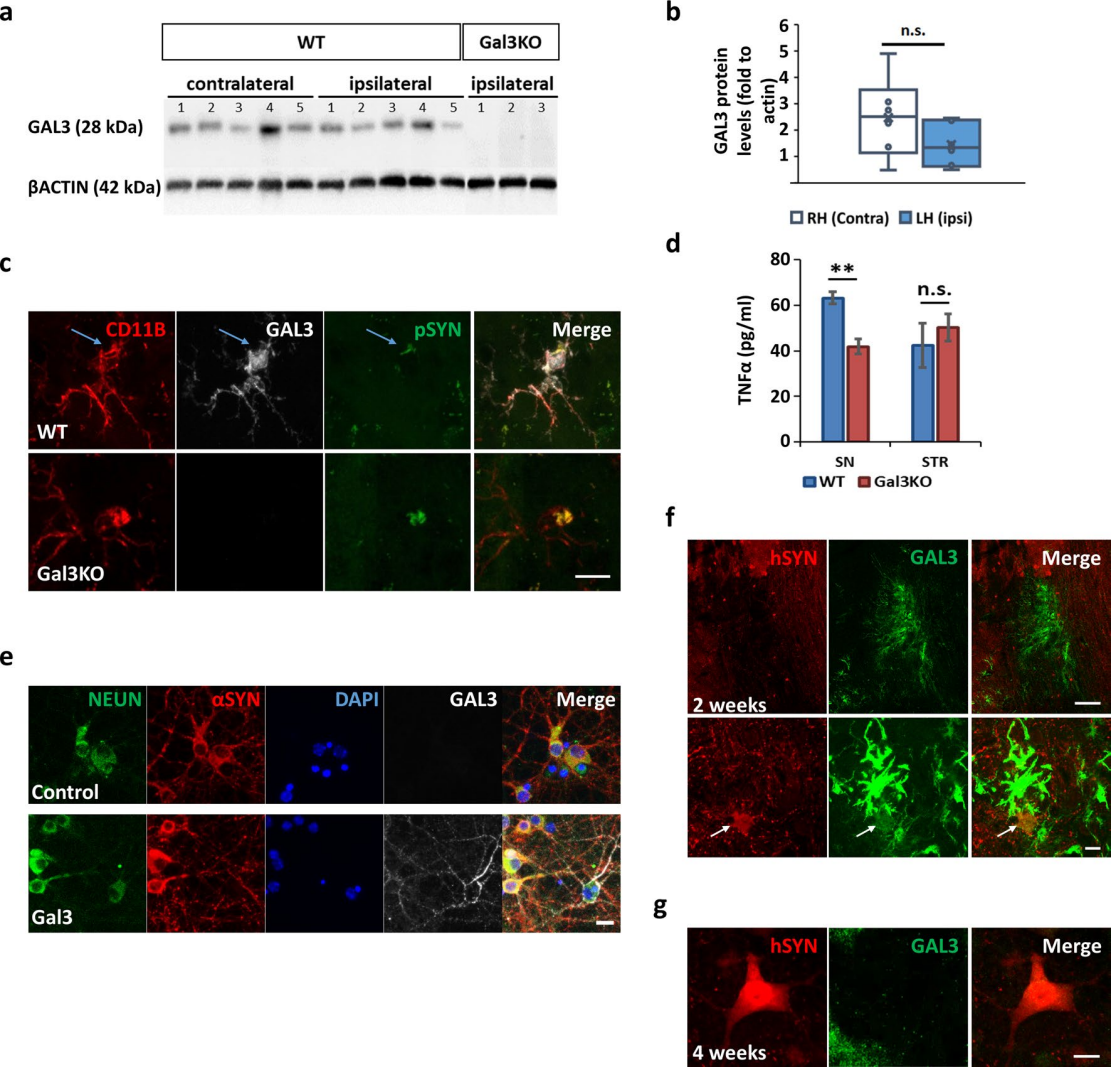
GAL3 early overexpression leads to chronic activation and neuronal internalization. **a** Western Blot against GAL3 from brain homogenates from WT and Gal3KO mice revealed constitutive expression of GAL3 in WT mice. **b** Western Blot quantification of total GAL3 protein in WT mesencephalon samples. No difference was found between contralateral (Right hemisphere, RH) and ipsilateral (Left hemisphere, LH) hemispheres. Data are expressed as percentage fold to actin. **c** Double immunofluorescence 6 months after adenovirus injection showed clusters of CD11B^+^ microglial cells highly reactive for GAL3. Internalized pSYN led to overexpression of GAL3 in WT microglia. pSYN was internalized by microglia independently of GAL3 genotype. Scale bar 20 µm. **d** TNFα quantification in SN and STR was performed on a MesoScale Discovery platform analysing brain extracts from AAV5-hSYN injected SN and STR (*p* < 0.05). **e** Neuronal primary cell culture from WT mice showed efficient Gal3 internalization after incubation with 0.8 µM gal3 for 10 days. Note no difference in endogenous αSyn staining after addition of Gal3. **f** hSYN/GAL3 double immunofluorescence from injection area of mice WT brains 2 weeks after injection revealed no colocalization and significant upregulation of GAL3. Scale bar 50 µm. GAL3^lo^/hSYN colocalization (white arrow) can be found near highly reactive GAL3 + cell indicating GAL3 release and neuronal GAL3 internalization. Scale bar 10 µm. **g** hSYN/GAL3 double immunofluorescence of mice WT brains 4 weeks after adenovirus injection revealed neuronal GAL3 staining. Scale bar 10 µm

The presence of GAL3 in the ventral mesencephalon is a prerequisite to sustaining its biological effects. To this end, we analysed the midbrain levels of GAL3 in WT mice after 6 months of hSYN overexpression by western blot. We found that GAL3 levels were not only upregulated compared with non-injected midbrains (Fig. 6a and b) but also demonstrated a basal constitutive expression of GAL3 in the ventral mesencephalon. Since reactive microglia are the cells supposedly expressing the highest levels of GAL3 [6, 10], we next performed tissue immunofluorescence against CD11B (highly expressed by microglia), GAL3 and pSYN in both WT and Gal3KO mice 6 months after the injection. Despite general low GAL3 reactivity within the SN, we were able to detect some highly reactive GAL3^+^ microglia in SN 6 months after hSYN overexpression (Fig. 6c). We were unable to find high GAL3^+^ cells other than in reactive microglia. Moreover, among the cytokines measured, the neurotoxic TNFα was found to be downregulated (Fig. 6d) in Gal3KO mice, suggesting a reduced chronic microglial activation. There is strong evidence of TNFα in the pathophysiology of PD [56], with elevated levels in PD patients [43].

Our group has previously identified a physical interaction of GAL3 with TREM2 in an AD in vivo model [7]. Consequently, we wondered if *Trem2* levels were altered in our PD model. mRNA analysis revealed a *Trem2* induction in animals overexpressing hSYN for 6 months (Supplementary Fig. 4a). Interestingly, Gal3KO failed to induce Trem2 expression, suggesting that *Trem2* induction is somehow related to the presence of GAL3. A similar effect was observed in the mRNA expression levels of *Mertk*, a myeloid receptor implied in the phagocytosis of apoptotic cells and related to GAL3 as it may act as an opsonin [11, 44]. Analysis of the transcription levels of *Mertk* revealed an induction in *Mertk* expression in WT animals injected with adenovirus overexpressing hSYN (Supplementary Fig. 4b), suggesting a possible phagocytic phenotype in microglia. In contrast, levels of *Mertk* mRNA were significantly lower in Gal3KO mice overexpressing hSYN revealing a significant effect of GAL3 deletion in *Mertk* expression. Taken together, our data suggest a chronically activated microglia phenotype in this PD model, which could be a local source of GAL3 for the surrounding neurons.

In light of our results from PD subjects showing an association of GAL3 to LB (Figs. 1 and 2), we wonder if dopaminergic neurons could internalise exogenous Gal3. We first treated neuronal cell lines (N27) and efficiently proved that Gal3 was internalised as soon as 3 h after incubation. Interestingly, in cells previously treated with PFF, the addition of exogenous Gal3 led to a specific Gal3 association with fibrillary structures (Supplementary Fig. 4c). Notably, no *Lgals3* mRNA was detected by RT-PCR in any condition upon addition of either Gal3, PFF, or both (data not shown). Later, we proved that primary neurons could incorporate exogenous Gal3 without affecting endogenous αSYN expression pattern (Fig. 6e). Finally, we analysed the brain 2 weeks after the injection and discovered a transient GAL3 release in the injection area of WT animals that were present 2 weeks after injection (Fig. 6f). This GAL3 release could have acted as a priming effector in our model, as suggested by low GAL3 immunostaining of some hSYN-positive cell bodies (Fig. 6f). Notably, 4 weeks after the injection, we were still able to detect some GAL3 inside neurons (Fig. 6g). In contrast, no overexpression of GAL3 was observed in the sourrounding area, indicating GAL3 presence inside the neuron after the transient overexpression. Our data support the hypothesis that microglia activation leads to GAL3 release that can be internalised by neurons where it can interact with αSYN fibrils; furthermore, basal GAL3 levels could be a sufficient source of GAL3 for neurons.

### Genetic deletion of GAL3 prevents neurodegeneration and motor impairment after overexpression of hSYN

**Fig. 7:**
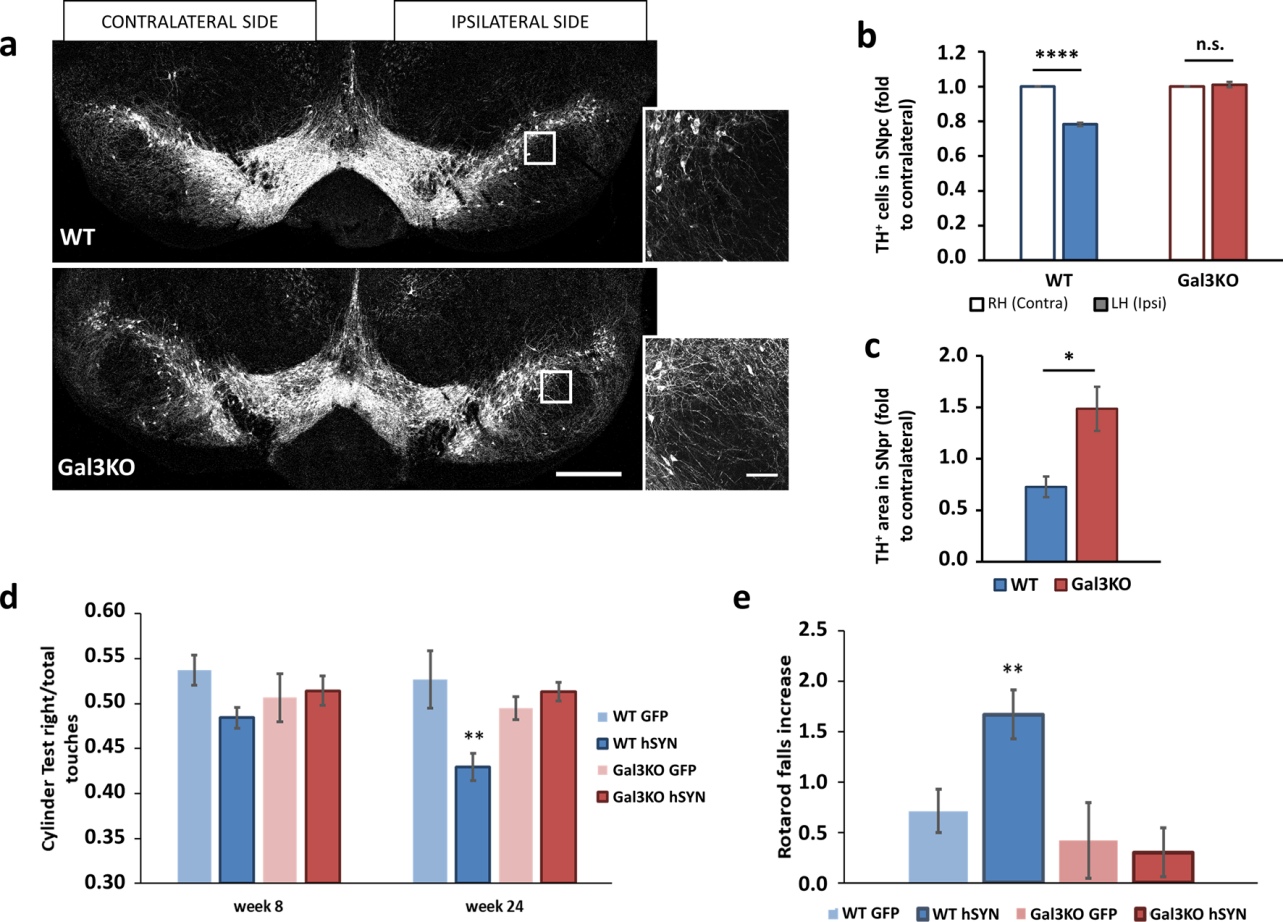
Gal3KO completely prevents neurodegeneration and protects from motor impairment. **a** Tyrosine hydroxylase (TH) immunostaining from whole mesencephalon, both adenovirus-injected ipsilateral hemisphere (right) and contralateral side (left) are shown. Note ipsilateral degeneration in WT but not in Gal3KO samples. Scale bar 500 µm. Amplified images represent the arborisation of ipsilateral SNpr for both genotypes. Note decreased arborisation in WT. Scale bar 100 µm. **b** TH^+^ stereological cell counting of the Substantia Nigra *pars compacta* (SNpc). Data are expressed as the left hemisphere (LH) fold to right hemisphere (RH) (*p* < 0.0001, *n* = 5). **c** Quantification of TH.^+^ area in the Substantia Nigra *pars* reticulata (SNpr) for dendritic tree arborisation. Dendritic arborisation was decreased in WT mice compared to Gal3KO mice. Data are expressed as ipsilateral area fold to contralateral side (*p* < 0.05, *n* = 5). **d** Mice were placed in a glass cylinder and allowed to explore. Number of times that mice touched the glass with each front paw was counted for a period of 5 min. Data are expressed as right paw touches per total touches. Test were performed at 8 and 24 weeks after adenoviral injection (*p* < 0.01, *n* = 10–14).** e** Mice were placed in a rotarod and challenged to constant acceleration. Number of falls was counted for each animal for a period of 5 min. Data are expressed as increase of individual data from 24 weeks after viral injection compared with individual data obtained from 8 weeks after injection (*p* < 0.01, *n* = 10–14)

To examine the potential role of GAL3 in neurodegeneration associated with overexpression of hSYN, immunohistochemical analysis of SN was combined with stereological techniques to quantify the number of dopaminergic neurons present in the SN of WT and Gal3KO mice (Fig. 7a). Since our approximation was based on ipsilateral injections, we could compare healthy uninjected SN with adenovirus-injected SN. We found that overexpression of hSYN for 6 months promotes specific neurodegeneration in dopaminergic cells of the SN. The loss of neurons was calculated to be 21.77% ± 0.92 of TH^+^ cells in the SN overexpressing hSYN. Interestingly, the deletion of GAL3 entirely prevented the αSYN-induced degeneration of nigral dopaminergic neurons. These animals showed complete preservation of the nigral dopaminergic system with around 100% survival rate of TH^+^ cells (Fig. 7b). Furthermore, the loss of neurons was accompanied by an apparent loss of dendritic processes in the SN of WT but not from Gal3KO animals (Fig. 7a). To quantify this observation, we measured the dopaminergic dendritic tree area in the SN *pars reticulata* (SNr). This analysis demonstrated that WT animals had about half of the TH^+^ area in the injected SN compared to the control SN (Fig. 7c). Consistent with the neuronal cell counting data, Gal3KO mice did not show any loss of TH^+^ staining. No neurodegenerative effect was observed after overexpression of GFP in WT or Gal3KO mice (Supplementary Fig. 5a).

Motor impairment is a clinical characteristic of PD; however, alteration of motor behaviour in mice models is variable and difficult to detect. In our study, we analysed motor behaviour 8 weeks and 6 months after the adenovirus injection. We did not find any difference in Open Field (Supplementary Fig. 5b), Grip Test or Corridor Test (data not shown), suggesting that motor deficits were not obvious but rather more specific. Given the ipsilateral nature of the AAV-hSYN injections, asymmetric motor behaviour was expected. To this end, we took advantage of the cylinder test, in which exploration instinct leads mice to touch the cylinder walls. Hence, an imbalance between the right and left paw touches is a signal of ipsilateral impairment [57]. Our results show how WT hSYN animals developed a clear ipsilateral imbalance 24 weeks after injection (Fig. 7d). As expected, AAV-GFP injected mice did not develop any imbalance, maintaining around 50% of touches with each paw. Importantly, this was also the case for the Gal3KO hSYN group. Supporting a better motor performance in Gal3KO than WT mice in response to AAV-hSYN injection, WT mice showed worsened motor coordination in the rotarod test 6 months after the injection compared with 8 weeks after the injection (Fig. 7e) while deletion of *Lgals3* gene prevented from motor instability and loss of balance and performed the test at similar levels to GFP injected mice.

Overall, our behavioural data confirmed motor impairment in WT animals overexpressing hSYN, most likely associated with specific dopaminergic degeneration. Importantly, GAL3 deletion entirely prevented motor abnormalities. Our study argues for a protective role of certain αSYN strains in the ventral mesencephalon against PD onset/pathogenesis, at least under conditions of hSYN overexpression, which has long been associated with PD aetiology [28, 35].

## Discussion

We have previously demonstrated a significant deleterious role of GAL3 in AD [7]. In this study, certain forms of the *LGALS3* gene were identified to increase the risk of AD, and, intriguingly, we showed the ability of GAL3 as a powerful amyloid beta binding agent [7]. More recently, we have established GAL3 as a critical pathological CSF biomarker of AD, strongly associated with other key AD CSF biomarkers [8]. GAL3 was further found to be inherently related to extracellular amyloid beta and intracellular tau aggregates. These findings suggest that GAL3 may exert a role in amyloid fibril formation, a pathological hallmark of human neurodegenerative diseases, including AD and PD. A causal role of GAL3 in PD is inferred from recent GWAS studies demonstrating that single nucleotide polymorphisms in the *LGALS3* gene are associated with an increased risk of PD [4]. Indeed, a study by Campbell and colleagues identified the presence of GAL3 as a corona in Lewy bodies (LB) in *post-mortem* samples from PD patients[20]. It should be considered that LB constitutes the tip of the iceberg, and, indeed, the evaluation of LB in the post-mortem brain does not always correlate with PD severity [15, 47]. The early stages of αSYN aggregation ultimately leading to LB, rather than LB themselves, are likely to be most detrimental for the neurons. Consequently, we investigated whether GAL3 is associated with LB in pathologies other than PD, like DLB. Furthermore, since the formation of pale bodies appears as a critical element preceding LB maturation [59], we evaluated the presence of GAL3 in both LB and Pale bodies in PD. We confirm and extend earlier findings, including that GAL3 is associated with LB in neuromelanin-containing cells from the SN of PD patients but not from control patients in *post-mortem* brain samples. However, GAL3 was not inherently related to all LB. The association between GAL3 and LB was not restricted to PD; thus, we demonstrate, for the first time, the presence of GAL3 within LB from *post-mortem* brain samples from DLB patients. Remarkably, GAL3 was intensively associated with Pale bodies, thus bringing the possibility that GAL3 could be causally related to PD by intervening in the early stages of LB formation.

We thoroughly investigated the mutual dynamics of the GAL3-αSYN interactions in PD patient samples. αSYN aggregation process has been described to follow several steps from intracytoplasmic punctate to the formation of an aggregation core, the development into pale bodies and finally, the formation of LB [38]. In this process, pale bodies are defined as non-symmetrical inclusions with homogenous to uniformly granular textures. Consistently with this definition, we observed different types of αSYN staining in *post-mortem* brain samples from PD patients. Pale bodies were found in the ventral mesencephalon as intense intracytoplasmic asymmetric inclusions, some surrounded by a less intense punctate halo. Notably, the beta-sheet marker Methoxy-XO4 efficiently discriminated between LB (Methoxy-XO4^+^) and pale bodies (Methoxy-XO4^−^). The study revealed that GAL3 was similarly associated with both LB and Pale Bodies. However, GAL3 was found to be more abundant in a specific type of LB with multiple dense cores. This finding provided evidence of a link between GAL3 and the morphology of LB. The loss of neuromelanin accompanied both LB and β-sheet negative accumulations compared with the control patients. Additionally, our results also showed that GAL3 was found in a puncta-like pattern according to the localisation of ruptured vesicles or the formation of small clusters. Strikingly, we found a precise negative correlation between GAL3 staining and the density of αSYN deposits, thus raising the potential role of GAL3 in the homeostasis of αSYN strains.

As previously stated, it has been demonstrated that GAL3 can bind to the interior side of lysosomal membranes upon membrane disruption due to its high glycosylation state [1], while LB are known to recruit several organelles, including lysosomes and mitochondria [53]. To this end, we have observed that GAL3 is present in lipofuscin-containing lysosomes in both control and PD patients. Lipofuscin accumulation is a well-known hallmark of ageing, when different lipid compounds are not adequately degraded and accumulate inside dysfunctional lysosomes. Indeed, applying high-resolution microscopy, we demonstrated that these vesicles condition the shape of LB in all *post-mortem* PD brains as they are not recruited to the LB. The importance of organelle recruitment in LB pathology was suggested by Shahmoradian and colleagues [53], demonstrating a dense population of vesicles and organelles in the interior of LB. Interestingly, lipofuscin-containing lysosomes are not incorporated into the LB in contrast with other organelles like mitochondria or autophagosomes that can be detected preferentially in the outer parts of the LB. Consequently, the recruitment of GAL3 to αSYN deposits could be viewed, at first glance, as a passive event in the process of LB formation. However, the possibility that GAL3 plays an active role in this process should not be discarded. Indeed, αSYN strains themselves have been found not to interact with membranes directly [60], and the possibility exists that endogenous molecules may act as a bridge between both elements. Thus, we then aimed at deciphering the potential role of Gal3 in αSyn dynamics by answering several important questions as follows: (i) does Gal3 have an affinity for αSyn strains?; (ii) does Gal3 have the ability to be internalized by dopaminergic neurons?; (iii) does Gal3 affect αSyn aggregation?; (iv) does Gal3 induce disaggregation of αSyn fibrils? and (v) does Gal3 play a significant role in neurodegeneration associated with αSyn? It is relevant to state that from a common αSyn polypeptide, multiple fibrillar conformers exhibiting differences in morphology, seeding capacity and toxicity can be generated [5, 27, 50]. To answer these questions, we took advantage of ThT aggregation assays along with in vitro culture of dopaminergic neurons in which the effects of Gal3 or truncated forms of the protein were tested.

Transcriptomic analysis of dopaminergic neuron has shown minimal *LGALS3* expression in the context of PD [32], and adult mouse brain [58]. However, GAL3 secretion has been demonstrated in brain cells like microglia [10], and GAL3 levels in serum have been detected to be increased in PD [13, 64], potentially acting as a source of GAL3 for neurons. We then measure the levels of GAL3 in frozen brain samples from PD patients showing that detectable GAL3 levels are found in the SN of PD patients. Moreover, levels of GAL3 in the cortex of these patients were significantly increased compared to control brains, suggesting that the availability of soluble GAL3 that neurons can internalise is higher in PD patients. In addition, the specific localisation of GAL3 in lipofuscin-containing vesicles could suggest an age-dependent internalisation of GAL3. Lipofuscin accumulation inside lysosomes can trigger lysosomal rupture [39]. A similar mechanism has been described for αSYN fibrils [9] that can expose internal lysosomal glycoproteins, thus promoting GAL3 binding. The lysosomal membrane then appears as the most probable location for αSYN-GAL3 interaction. Interestingly, GAL3 increased levels can be triggered by multiple factors in the CNS and/or the periphery. Some of these are considered as risk factors for PD, like obesity, diabetes or inflammation [42]; our work then establishes a molecular mechanism that can relate these factors with PD pathogenesis. In that line, we proved that dopaminergic cell lines and primary mouse neurons can internalise exogenous Gal3, which is rapidly associated with any previous αSyn accumulation. In contrast, no endogenous GAL3 expression was detected in response to αSyn. Thus, exogenous internalisation of GAL3 arises as a feasible mechanism for GAL3 accumulation. In our mouse model, GAL3 basal expression was detected after 6 months which could be sufficient for neuronal internalisation. Furthermore, we proved a marked microglial GAL3 overexpression in the first days after injection supported by local expression of GAL3 in response to internalised phosphorylated αSYN. While it is likely that the presence of GAL3 in dopaminergic neurons is derived from microglia rather than neurons, we were unable to definitively identify the source. Microglia is known to overexpress and release GAL3 in response to various stimuli and neurodegenerative conditions [22]. However, we did not observe any GAL3-overexpressing cells in the vicinity of LB-containing neurons in humans, and only a small number of microglia were found in the mouse model. Still, it is possible that microglia or other cell types could produce and secrete low levels of GAL3 that are undetectable by immunofluorescence techniques but later accumulate inside the neurons, during decades of disease development. Indeed, our findings of effective neuronal internalization of GAL3 not only opens up new possibilities, but also suggest a potential mechanism that may be relevant for future research.

Once we demonstrated the ability of Gal3 to efficiently bind to αSyn fibrils and the intrinsic ability of dopaminergic neurons to internalise Gal3, we sought to analyse if Gal3 affects aggregation/disaggregation of αSyn. ThT aggregation studies demonstrated that Gal3 impairs the de novo formation of fibrils, limiting the fibrillation of monomeric αSyn in solution. This effect was proved to be dependent on Gal3 via its CRD. Interestingly, we tested if Gal3 could catalyse the disaggregation of fibrils into αSyn monomers. However, no decrease in ThT fluorescence was observed. Instead, and remarkably, we found that Gal3 modifies pre-formed fibrillary structures by binding to them and further destabilising the fibrillary network, including releasing smaller fibrillary species. Fibrils changed their conformation in response to Gal3, which was detected by ThT but not by ANS dye, revealing no change in hydrophobicity. Proteinase K digestion of fibrils after incubation with Gal3 revealed that, in presence of monomeric αSyn, the addition of Gal3 induces higher susceptibility to Proteinase K digestion compared with the control condition. However, this effect was lost in a context of high fibrilization using preformed fibrils, where the addition of Gal3 did not promote a higher susceptibility to Proteinase K digestion. We can then speculate that Gal3 interaction with αSyn leads to soluble species release but the core of the fibrils remains insoluble. Further spectroscopic analysis (OPTIR) showed a reduction of the intensity corresponding to beta-sheet structures that could be explained by a decrease in ThT intensity and morphological changes observed in EM micrographs. This suggested a direct interaction between αSyn and Gal3. To undoubtedly demonstrate this possibility, we examined the binding affinity between the two proteins by ELISA, showing that monomeric and fibrillar αSyn selectively bind Gal3. Numerous studies have reported the importance of smaller aggregating species of amyloid β (Aβ), tau, αSyn and several other amyloid proteins in disease initiation/progression [23, 29, 52]. Indeed, the position and size of αSyn strains within the cell are believed to be crucial for their mode of toxicity. For instance, the size of the strains has been shown to be critical to trigger cellular toxicity through their β-sheet core and solvent-exposed hydrophobic surfaces [24]. Long fibrils are, in nature, inert or less reactive than short fibrils. Indeed, long fibrils have been shown to be less toxic than short fibrils, an effect likely associated with their lower proportion of fibrillar ends at the same mass concentration. Recently, PD patients have been shown to have more proportion of short and soluble αSYN fibrils [18]. In addition, short fibrils are known to induce PD-like pathology by spreading when injected into the brains of unlesioned animals [12, 17]. Consequently, we aimed to test the toxicity of our resulting fibrils in a dopaminergic cell line. We observed increased toxicity after adding the αSyn-Gal3 combination after 48 h of treatment compared with unmodified αSyn treatment, while the size and number of deposits were not affected. This finding confirms Gal3 as an endogenous molecule with the ability to bind and modify αSyn strains leading to increase toxicity in vitro, anticipating an important role of Gal3 in PD.

The critical question that arises from the in vitro experiments is the biological significance of these findings. To achieve this goal, we took advantage of the unilateral AAV-hSYN model mice, which allows long-lasting expression of hSYN in dopaminergic neurons along with dopaminergic neurodegeneration and asymmetric motor behaviour [61]. Consequently, we overexpressed hSYN in ventral mesencephalic dopaminergic neurons from WT and Gal3KO mice to test the role of GAL3 in αSYN aggregation and subsequent dopaminergic neurodegeneration and motor impairment. As a first step, we validated the model detecting hSYN in the ventral mesencephalon with high sensitivity for dopaminergic neurons as early as 2 weeks after the injection that went through anterograde transport to the striatum as evidenced by a robust hSYN staining. Notably, hSYN was expressed at high levels in the nigrostriatum for at least 6 months, when most analyses were performed. Thus, we validated the usefulness of the model to analyze, among others, the effect of GAL3 deletion in αSYN aggregation and how a potential impact on αSYN aggregation affects dopaminergic integrity and asymmetric motor behaviour. Strikingly, analysis of αSYN strains demonstrated that Gal3KO mice presented evident rounded intracytoplasmic inclusions of αSYN in the ventral mesencephalon resembling human LB. This finding is in agreement with our in vitro experiments and points to GAL3 as an endogenous molecule playing a decisive role in the aggregation and shaping of αSYN inclusions.

The next obvious question that arises is the potentially toxic nature, or not, of these strains in comparison with those found in WT mice. Indeed, it has been long suggested that oligomers rather than αSYN fibrils are the neurotoxic species [14, 54]. Interestingly, despite an increased number of dopaminergic neurons presented hSYN accumulation within their soma in Gal3KO compared to WT, Gal3KO dopaminerfic system was fully preserved, supporting the idea that αSYN accumulation is not toxic per se but rather a mechanism for cell survival. An event that is supposed to play a critical role is the post-translational modification of αSYN at Ser129, and indeed, 90% of αSYN deposition in LB is phosphorylated at this residue [63]. We found no significant change in the degree of phosphorylation at Ser129. These findings are in line with observations from Vekrellis and colleagues, who have recently discovered that Ser129 phosphorylation is not needed for fibril formation and seeding effect in mice while increasing neurotoxicity [33]. Recently, Burbidge and colleagues demonstrated that GAL3 mediates αSYN secretion through lysosomal rupture [9], suggesting that GAL3 contributes to both propagation and toxicity. Similarly, we detect signs of chronic ipsilateral microglia activation only in WT mice characterised by an increase in the secretion of neurodegenerative cytokine Tnfα and the genetic expression of some DAM receptors like *Trem2* and the phagocytic receptor *Mertk*. All this suggests that αSYN-GAL3 interaction triggers a vicious cycle that includes the formation of smaller species, increased secretion and chronic microglia activation that could be maintained in the long term to provoke progressive neurodegeneration.

To undoubtedly demonstrate the detrimental role of GAL3 in αSYN aggregation and associated toxicity, we then analysed the effect of hSYN overexpression on the integrity of the dopaminergic system. Stereological cell counting of SN dopaminergic neurons showed a consistent 25% loss of nigral neurons in WT mice which was completely prevented in Gal3KO animals, demonstrating the less toxic nature of αSYN strains lacking GAL3. It has been estimated that motor impairments can be detected after 30–60% of dopaminergic loss in humans [36]; thus, our mouse model mimics the earliest symptomatic stages of PD. Indeed, we failed to detect differences in open field parameters, thus suggesting that overall motor behaviour was similar between genotypes with no major locomotor impairments or anxiolytic behaviour. However, the unilateral nature of the injection of AAV makes tests relying on asymmetry in motor behaviour more accurate for detecting these motor disturbances [61]. To this end, we performed the cylinder test, demonstrating that WT hSYN but not Gal3KO animals developed a clear ipsilateral imbalance 6 months after injection. The rotarod test corroborated better motor performance in Gal3KO mice than in WT mice.

Taken together, we conclude that GAL3 is an endogenous molecule expressed in the ventral mesencephalon, highly associated with pathological αSYN strains, including Pale bodies and LB. We provide evidence that Gal3 has a high affinity to αSyn. Importantly, it acts as a critical modifying factor for αSyn aggregation, enabling modulation of shape, growth, and toxicity of pathological αSyn strains. The predicted important role of GAL3 in αSYN pathology is supported by recent GWAS studies that have identified *LGALS3* as a PD risk gene [4]. We have earlier identified that *LGALS3* is also an AD risk gene [7], and we and others have demonstrated the ability of Gal3 to affect the aggregation of amyloid beta, a distinguished hallmark of the disease [7]. Consequently, GAL3 emerges as a decisive endogenous factor regulating amyloid deposition in the diseased brain, a hallmark of most important neurodegenerative disorders. Pharmacological targeting of GAL3 appears as a promising preclinical strategy to combat PD-associated αSYN pathology.

## Supplementary Information

Below is the link to the electronic supplementary material.

Supplementary file1 (PDF 2895 KB)

## Data Availability

All data supporting the findings of this study are available within the paper and its Supplementary Information. Extra information about the data is available from the corresponding author upon reasonable request.
