# Supplementary material for: Galectin-3 shapes toxic alpha-synuclein strains in Parkinson’s disease
Source: Acta Neuropathol. 2023 May 18;146(1):51–75. doi: 10.1007/s00401-023-02585-x (PMC10261194; doi:10.1007/s00401-023-02585-x)
Supplement: Supplementary file 1 — Supplementary file1 (PDF 2895 KB) [file 401_2023_2585_MOESM1_ESM.pdf]

Supplementary Figure 1.

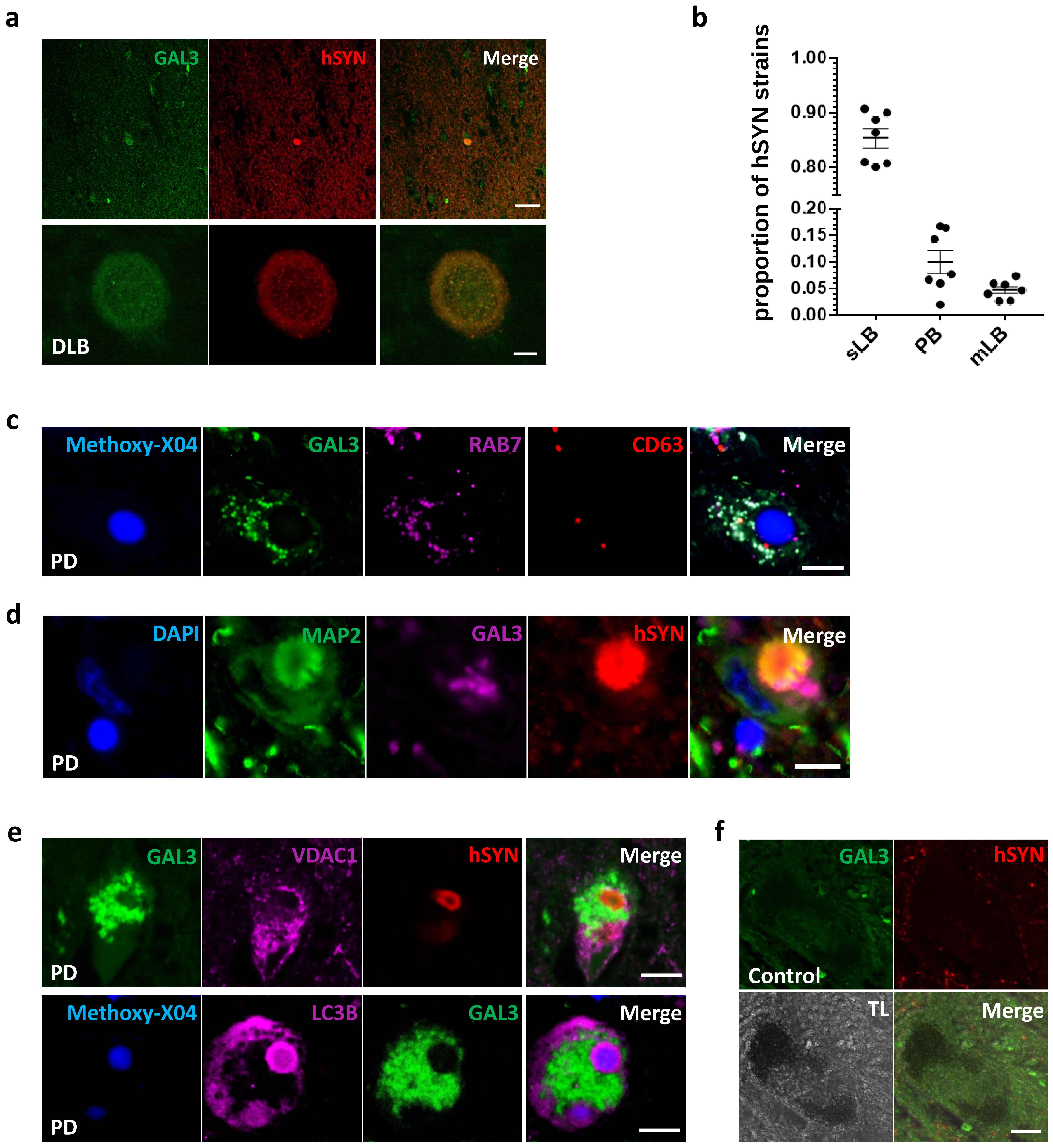

Supplementary Figure 2.

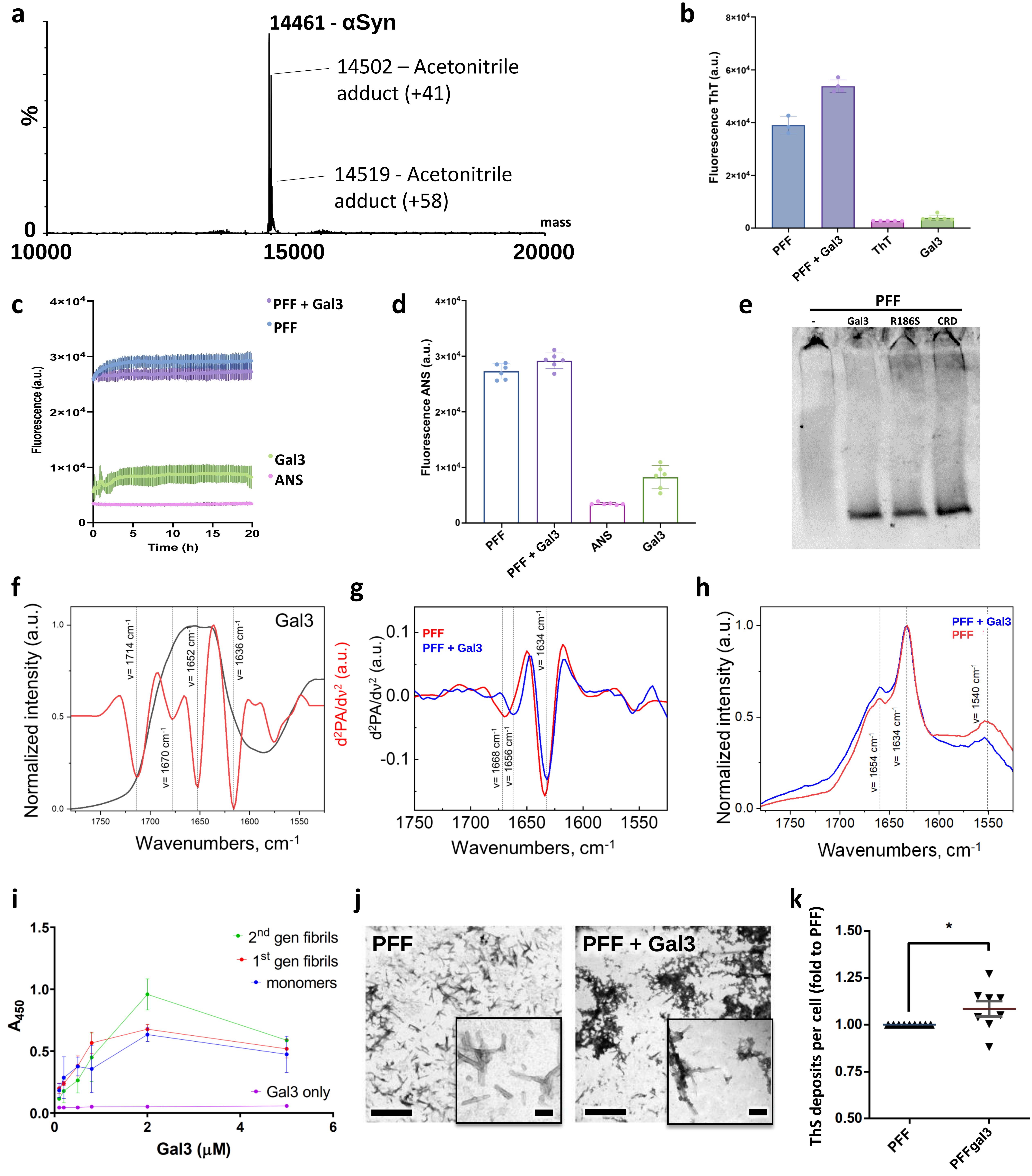

Supplementary Figure 3.

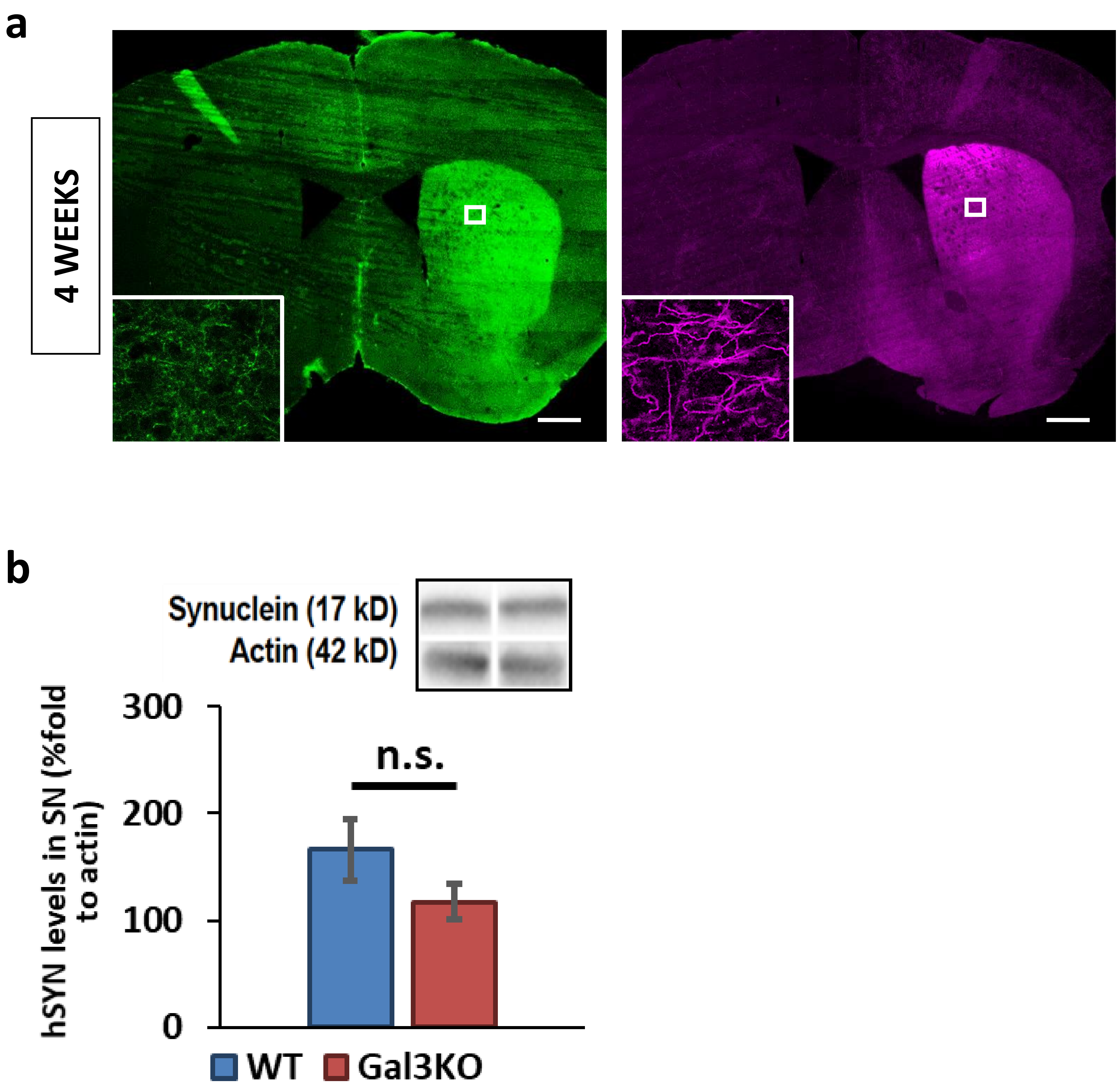

Supplementary Figure 4.

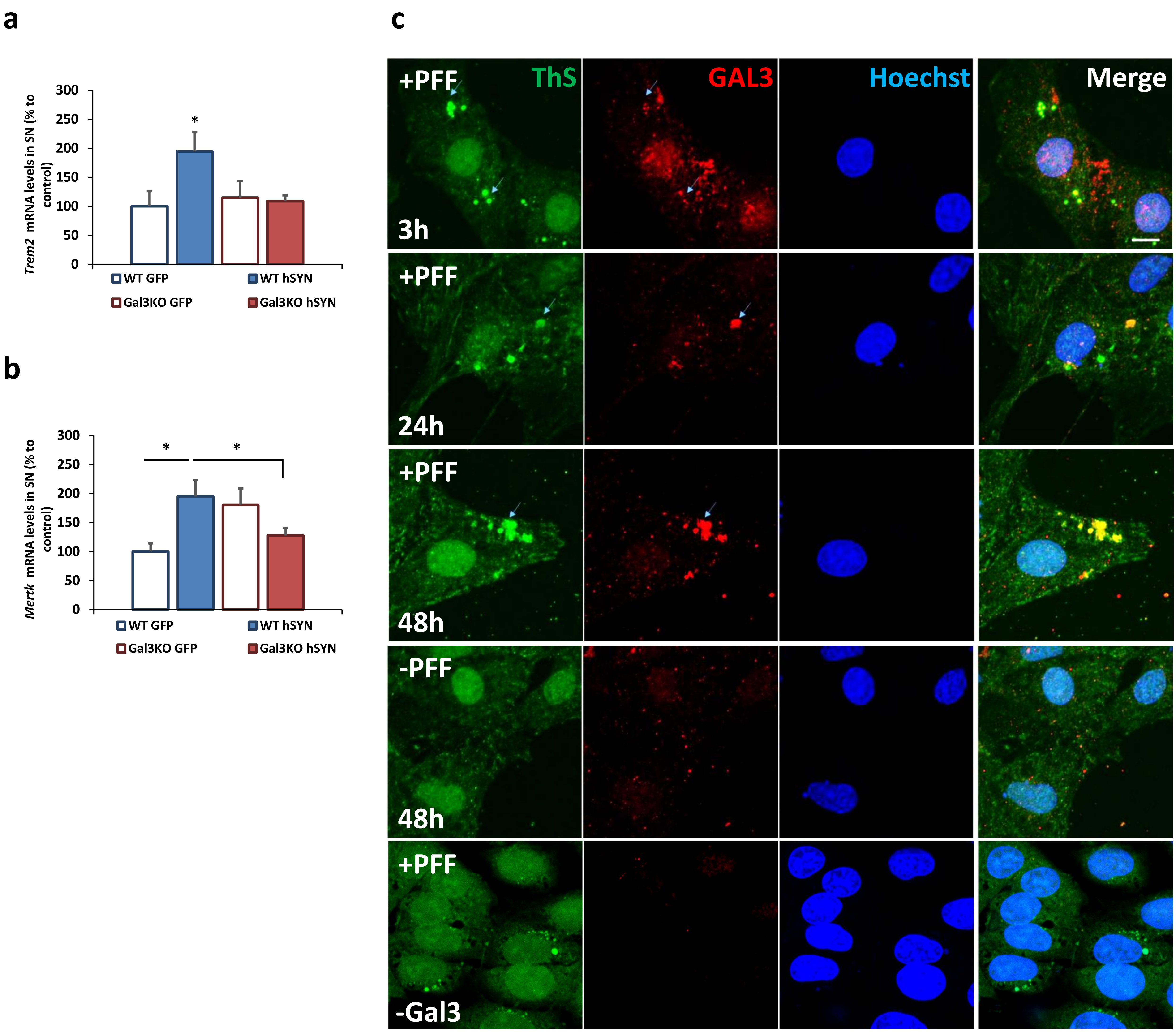

Supplementary Figure 5.

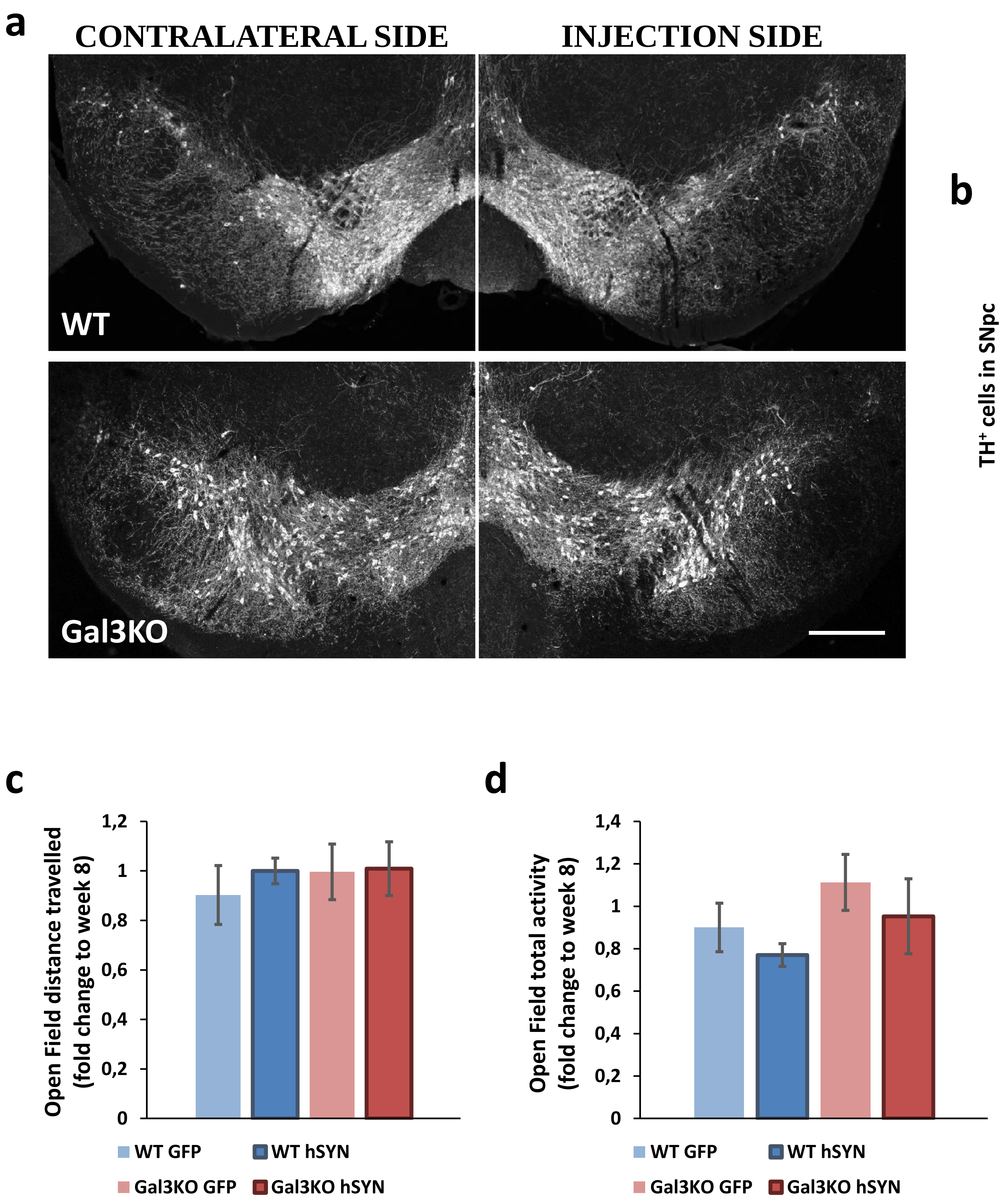

Supplementary Figures Legend

Supplementary Figure 1

a) Colocalisation of Galectin-3 (GAL3) in α-synuclein deposits in DLB patients. Human DLB immunostaining against GAL3 and the human form of αSyn (hSYN). GAL3 is present in Lewy Body (LB) in pathological conditions. Scale bar upper panel 50 µm. Scale bar lower panel 5 µm. **b)** Distribution of hSYN strains in PD samples studies. Methoxy-X04 was used as a specific marker of LB. Single (sLB) and multiple core LB (mLB) were discriminated. **c)** GAL3 colocalises with lysosome-endosome marker (RAB7) but not with exosome marker CD63 around Lewy Body from PD patient. Scale bar 10 µm. **d) I**mmunofluorescence analysis revealed that GAL3 accumulates inside MAP2 neurons in the vicinities of LB. Scale bar 10 µm. **e)** GAL3 does not colocalise with mithocondrial marker VDAC1 nor autophagosome marker LC3B which are recruited to the LB. Scale bar 10 µm. **f)** GAL3 is not present in neuromelanin vesicles in dopaminergic neurons from control patients. Scale bar 10 µm**.**

Supplementary Figure 2

a) Electrospray Liquid chromatography (ES-LC) mass spectroscopy confirmed the expected mass of recombinant αSyn to 14461 daltons. The additional peaks of +41 and +58 are expected adducts from the acetonitrile used for in the procedure. **b)** Thioflavin T (ThT) aggregation assay was performed in presence of fully aggregated pre-formed fibrils (PFF) and recombinant galectin-3 (Gal3). Quantification at 15h from Figure 3c. **c)** ANS aggregation assay was performed in presence of PFF and Gal3. Gal3 does not alter ANS signal from PFF. **d)** Quantification at 15h from c). **e)** Native PAGE Western Blot of the final results obtained after overnight incubation of PFF with distinct forms of Gal3. Note that all forms of Gal3 promoted an increase in smaller soluble species. **f)** OPTIR spectra (black) and second derivative calculation (red) of soluble gal3. **g)** Second derivative calculation of fibrils after 16h incubation with Gal3. Note 1668 and 1634 cm^{-1} peaks shifted to the right after incubation with Gal3, note decreased signal at 1634 cm^{-1} wavelength in PFFgal3 samples corresponding to lower number of β-sheet structures. **h)** OPTIR spectra of samples normalized to the maximum intensity peak. **i)** Direct interaction of Gal3 with different αSyn species was investigated by ELISA. Increasing Gal3 concentration were pre-coated in a 96 well plate and 2 µM αSyn species were incubated. 450 nm absorbance was measure to detect bounded protein. **j)** Effect of sonication on PFF and PFF pre-incubated with Gal3 for 16h. 5 pulses at 0.5 on/off cycles and 30% power was applied. Scale bar 1 µm. Scale bar of amplified image 100 nm. **k)** Addition of sonicated PFF pre-incubated with Gal3 for 30 minutes to dopaminergic cell line N27 for 48h provoked an increased number of insoluble deposits per cell compared with non-incubated PFF. Deposits were identify based on thioflavin-S (ThS) immunoreactivity, $p<0,05$.

Supplementary Figure 3

a) Effective expression of GFP and hSYN proteins in striatal terminals was observed 4 weeks after the injection confirming an anterograde transport of the protein. Scale bar 500 µm. Scale bar of amplified images 20 µm. **b)** Western Blot quantification of total hSYN protein in the mesencephalon of injected mice revealed no difference in expression of the protein within genotypes.

Supplementary Figure 4

a) Expression analysis of *Trem2* and *Mertk* in whole brain sections. Trem2 expression is significantly elevated in WT mice overexpressing hSYN (WT hSYN) but no change was observed in Gal3KO hSYN group, $p<0.05$. **b)** *Mertk* expression is significantly elevated in WT hSYN group, and present an inverse response in Gal3KO mice with increased levels in the control group and decreased levels in Gal3KO hSYN group indicating a dysregulation of *Mertk* in comparison with WT genotype, $p<0.05$. **c)** N27 cell line treated for 48h with PFF (1µg/ml) and, simultaneously, with 50 nM Gal3 for 3, 24 or 48 hours. Immunostaining against Gal3 and αSyn revealed a time dependent specific interaction of Gal3 and αSyn after only 3 hours that is maintained over time. Scale bar 10 µm.

Supplementary Figure 5

a) TH immunostaining of the SN showing GFP injected side (ipsilateral, right panel) and non-injected side (contralateral, left panel). No apparent loss of dopaminergic neurons was observed. Scale bar 500 µm. **b)** TH+ stereological cell counting of the SNpc (n=3). Data are expressed as left hemisphere (LH) fold to right hemisphere (RH). **c)** Open Field distance travelled. Total distance (cm) travelled by mice was measure using SMART® software. Data is express as fold change between 24 weeks data and 8 weeks data. No difference was observed during the experiment, n=10. **d)** Open Field motor activity. Total activity (cm^2/s) of mice was measure using SMART® software. Data is express as fold change between 24 weeks data and 8 weeks data. No difference was observed during the experiment, n=10.
